# Direction and magnitude of cerebrospinal fluid flow vary substantially across central nervous system diseases

**DOI:** 10.1186/s12987-021-00251-6

**Published:** 2021-04-01

**Authors:** Per Kristian Eide, Lars Magnus Valnes, Erika Kristina Lindstrøm, Kent-Andre Mardal, Geir Ringstad

**Affiliations:** 1 Deptartment of Neurosurgery, Oslo University Hospital-Rikshospitalet, Nydalen, PB 4950, 0424 Oslo, Norway; 2 Institute of Clinical Medicine, Faculty of Medicine, University of Oslo, Oslo, Norway; 3 Department of Mathematics, Faculty of Mathematics and Natural Sciences, University of Oslo, Oslo, Norway; 4 Institute for Cancer Genetics and Informatics, Oslo University Hospital, Oslo, Norway; 5 Department of Numerical Analysis and Scientific Computing, Simula Research Laboratory, Oslo, Norway; 6 Department. of Radiology, Oslo University Hospital-Rikshospitalet, Oslo, Norway

**Keywords:** Cerebrospinal fluid, Cerebral aqueduct, Craniocervical junction, Flow direction, Flow magnitude

## Abstract

**Background:**

Several central nervous system diseases are associated with disturbed cerebrospinal fluid (CSF) flow patterns and have typically been characterized in vivo by phase-contrast magnetic resonance imaging (MRI). This technique is, however, limited by its applicability in space and time. Phase-contrast MRI has yet to be compared directly with CSF tracer enhanced imaging, which can be considered gold standard for assessing long-term CSF flow dynamics within the intracranial compartment.

**Methods:**

Here, we studied patients with various CSF disorders and compared MRI biomarkers of CSF space anatomy and phase-contrast MRI at level of the aqueduct and cranio-cervical junction with dynamic intrathecal contrast-enhanced MRI using the contrast agent gadobutrol as CSF tracer. Tracer enrichment of cerebral ventricles was graded 0–4 by visual assessment. An intracranial pressure (ICP) score was used as surrogate marker of intracranial compliance.

**Results:**

The study included 94 patients and disclosed marked variation of CSF flow measures across disease categories. The grade of supra-aqueductal reflux of tracer varied, with strong reflux (grades 3–4) in half of patients. Ventricular tracer reflux correlated with stroke volume and aqueductal CSF pressure gradient. CSF flow in the cerebral aqueduct was retrograde (from 4th to 3rd ventricle) in one third of patients, with estimated CSF net flow volume about 1.0 L/24 h. In the cranio-cervical junction, net flow was cranially directed in 78% patients, with estimated CSF net flow volume about 4.7 L/24 h.

**Conclusions:**

The present observations provide in vivo quantitative evidence for substantial variation in direction and magnitude of CSF flow, with re-direction of aqueductal flow in communicating hydrocephalus, and significant extra-cranial CSF production. The grading of ventricular reflux of tracer shows promise as a clinical useful method to assess CSF flow pattern disturbances in patients.

**Graphic abstract:**

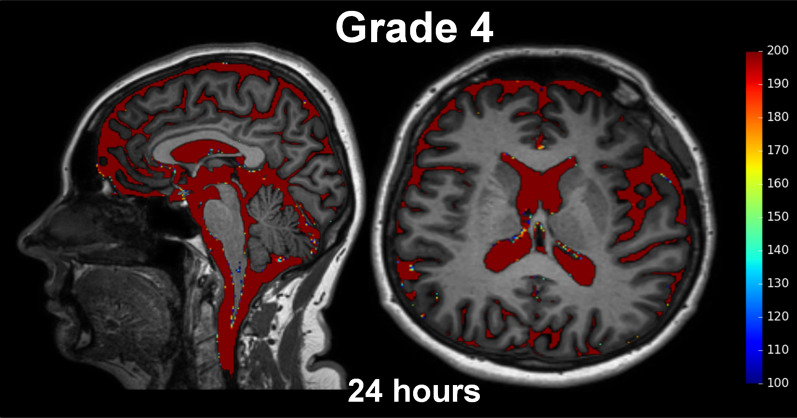

**Supplementary Information:**

The online version contains supplementary material available at 10.1186/s12987-021-00251-6.

## Introduction

Over the last few years, the impact of cerebrospinal fluid (CSF) homeostasis for brain function has gained renewed interest, not least facilitated by the description of the glymphatic system in 2012 [1], and the meningeal lymphatic vessels in 2015 [2, 3]. These discoveries have highlighted the role of CSF in central nervous system (CNS) metabolic function and immune surveillance [4], and the role of CSF for removal of metabolic waste products from the brain in neurodegenerative and dementia diseases [5]. The CSF production and flow patterns are among several factors that may affect glymphatic and meningeal lymphatic functions, but remain less characterized.

Recently, several reviews have addressed the lack of consensus regarding magnitude and direction of CSF flow in man [4, 6–9]. A traditional concept of CSF circulation was coined by Harvey Cushing in 1925 as “The third circulation” (first referring to blood and second to lymphatic circulation) [10]. According to this view, CSF is thought to be mainly produced by the choroid plexus and circulate from the lateral and third ventricles to the fourth ventricle and further to the surface of the brain with efflux to venous blood via arachnoid granulations. This view has been challenged [6, 8]. Traditionally, the CSF production rate has been considered about 0.30–0.40 ml/min, i.e., about 0.5 L/24 h, with 80% of CSF being produced within the choroid plexus [4], but the methods upon which the numbers are based are debated [6]. Others have provided experimental evidence that there is no CSF production within the choroid plexus, and that there is no “circulation” or unidirectional CSF net flow within e.g., the cerebral aqueduct [8, 11]. In vivo human evidence has been given that the choroid plexus may also well function as an efflux route for molecules [12]. Therefore, a broader insight regarding characteristics of CSF flow in man is highly needed, particularly in brain diseases associated with CSF disturbances.

This study examined patients with different CSF disorders with regard to how CSF tracer moves from the subarachnoid space into cerebral ventricles, and the direction and magnitude of CSF flow in the cerebral aqueduct and at the cranio-cervical junction. Two magnetic resonance imaging (MRI) modalities were used, namely dynamic intrathecal contrast-enhanced MRI with an MRI contrast agent serving as a CSF tracer for assessment of long-term flow patterns, and phase-contrast MRI, which assesses CSF flow velocity and direction through a much narrower window in time and space. The observations disclose marked variation between patient categories concerning retrograde transport of tracers from subarachnoid spaces to supra-aqueductal ventricles (referred to as ventricular reflux of tracer), and in the direction and magnitude of CSF flow in cerebral aqueduct and cranio-cervical junction. Retrograde CSF flow from 4th to 3rd ventricle was typical for communicating hydrocephalus. Moreover, the present results suggest that the MRI-derived quantitative data may be described by qualitative assessment, using a grading system for supra-aqueductal ventricular tracer reflux that is feasible in daily clinical context [13].

## Methods

### Approvals and patients

This study was approved by the Regional Committee for Medical and Health Research Ethics (REK) of Health Region South-East, Norway (2015/96), the Institutional Review Board of Oslo university hospital (2015/1868) and by the National Medicines Agency (15/04932-7).

The study included consecutive patients undergoing intrathecal contrast-enhanced MRI and phase-contrast MRI, as part of their neurosurgical work-up of various CSF disorders within the Department of Neurosurgery at Oslo University Hospital, Norway during the period October 2015 to June 2018. They were included after written and oral informed consent. The study was conducted according to ethical standards according to the Helsinki Declaration of 1975 (and as revised in 1983). Exclusion criteria were either a history of hypersensitivity reactions to contrast media agents, a history of severe allergy reactions in general, evidence of renal dysfunction (i.e., having normal glomerular filtration rate, GFR), age < 18 or > 80 years, pregnant or breastfeeding women.

### MRI acquisitions and analysis

The MRI protocol has previously been described [14, 15], and followed a standardized protocol. MRI acquisitions were obtained using a 3T Philips Ingenia MRI scanner (Philips Medical systems, Best, The Netherlands).

### MRI biomarkers of CSF space anatomy

T1-weighted MRI was used for assessment of CSF space anatomy. We used axially reconstructed images (1 mm thickness) for estimation of Evans’ index (the largest diameter of the frontal horns divided by the largest inner diameter of the cranium in the same slice) [16]. Coronal images were used to assess the callosal angle (the angle between lateral ventricles at the level of the posterior commissure) [17]. Coronal images were used to assess the presence of disproportionately enlarged subarachnoid space hydrocephalus (DESH; characterized by a combination of enlarged ventricles, widening of Sylvian fissure, and tight upper convexities) [18].

### Dynamic intrathecal contrast enhanced MRI

The protocol used for intrathecal contrast enhanced MRI has previously been described in detail [14, 15]. Identical imaging protocol settings were used before and at different time points after intrathecal injection of gadobutrol (0.5 mmol of 1.0 mmol/ml gadobutrol, Gadovist, Bayer Pharma AG, Berlin, Germany).

With regard to image analysis, the FreeSurfer software (version 6.0) (http://surfer.nmr.mgh.harvard.edu/) was used for segmentation, parcellation and registration/alignment of the longitudinal data to assess the increase of T1 intensity due to CSF tracer within the cerebral ventricles; for methods description, see [19]. For every segmented ventricular area, we computed the median T1 signal unit for each time point, and divided the median signal unit against the signal unit of a reference ROI placed within the orbital fat tissue in axially reconstructed images from the same T1 volume scan. This signal unit ratio refers to the normalized* T1 *signal units and adjusts for the baseline image grey scale changes between scans.

### Grading of ventricular reflux

**Fig. 1:**
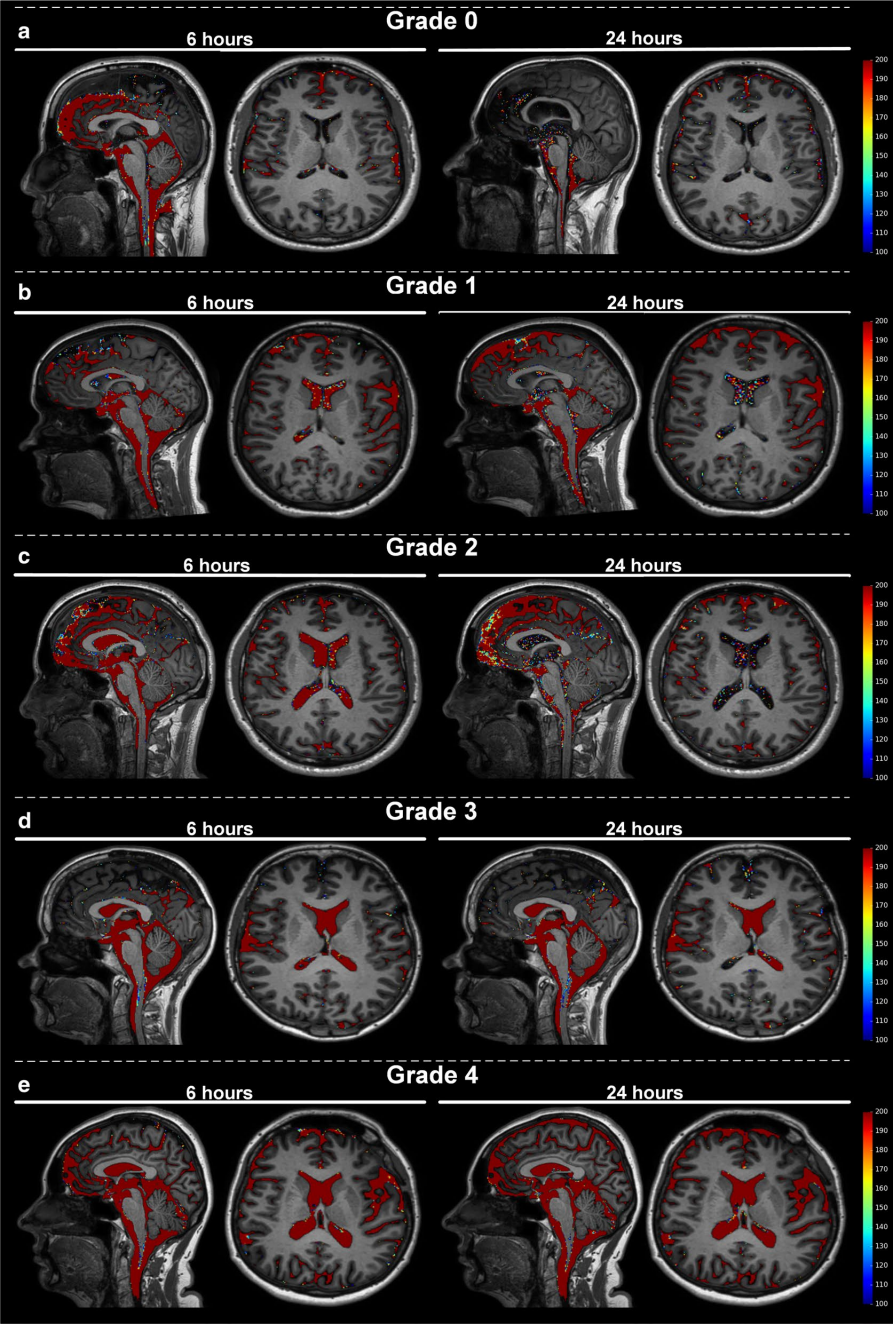
The degree of enrichment of tracer within supra-aqueductal ventricles illustrates the different grades of ventricular reflux. Ventricular reflux was categorized in five categories: (**a**) Grade 0: No signs of supra-aqueductal reflux of tracer. **b** Grade 1: Sign of supra-aqueductal reflux of tracer. **c** Grade 2: Transient enrichment of lateral ventricles about 6 h after tracer administration. **d** Grade 3: Lasting enrichment of lateral ventricles, but not isointense with subarachnoid CSF, examined about 24 h after tracer administration. **e** Grade 4: Lasting enrichment of lateral ventricles Day 2, with isointense T1 signal in ventricles and subarachnoid CSF after about 24 h. The color scale to the right shows the percentage change in signal unit ratio, determined by FreeSurfer software. Notably, signal change within the parenchyma is extracted by FreeSurfer in order to only present the percentage signal change within the CSF spaces of the ventricles and the subarachnoid space

The degree of ventricular reflux of CSF tracer was assessed by visual inspection of T1 weighted images, as recently described [13], and further detailed in Fig. 1 showing tracer enrichment in CSF spaces segmented using FreeSurfer.

### Phase-contrast MRI at level of cerebral aqueduct and cranio-cervical junction

**Fig. 2:**
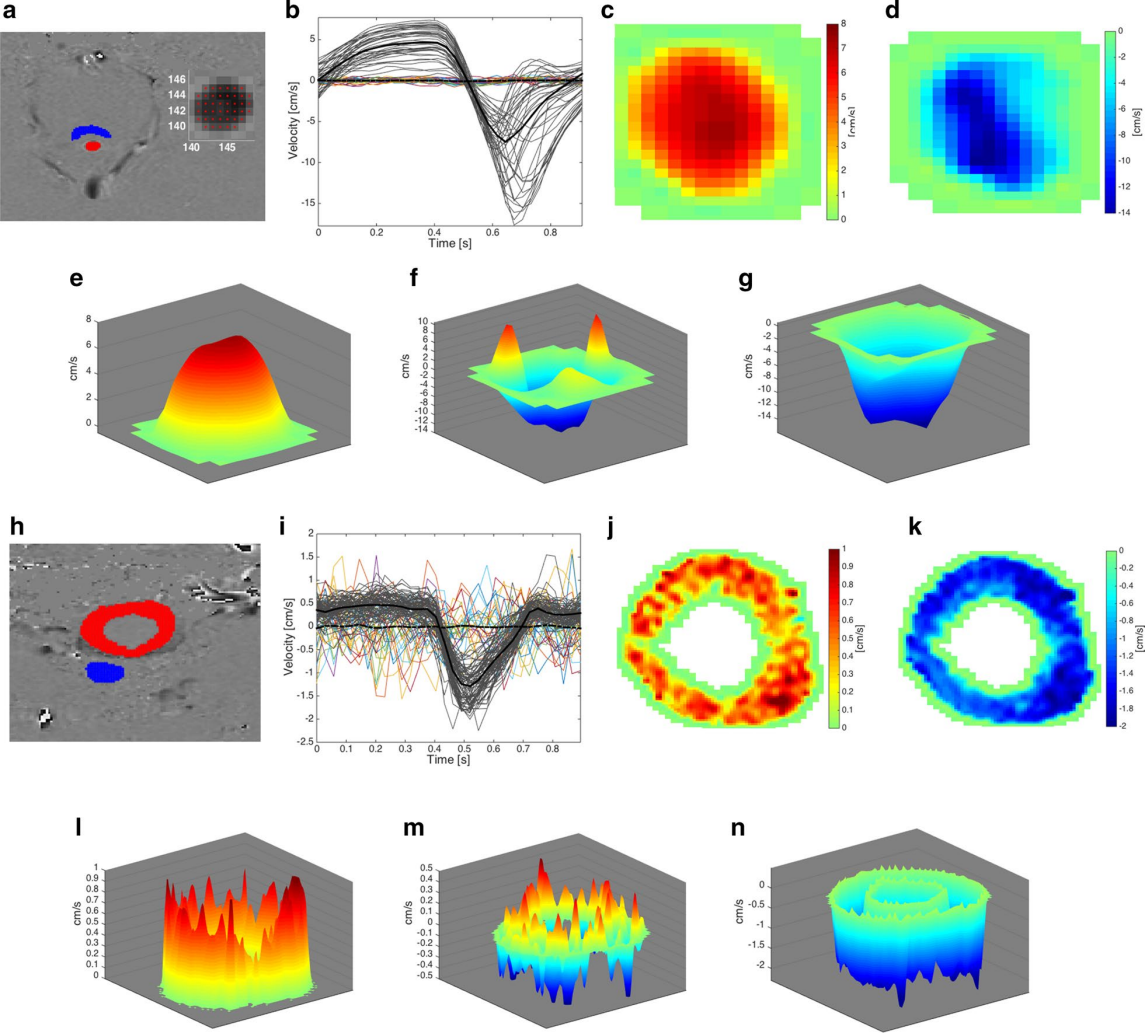
Phase-contrast MRI of the cerebral aqueduct (**a**–**g**) and cranio-cervical junction (**h**–**n**) in a 75 years old male individual with iNPH. Cerebral aqueduct: (**a**) The ROI (0.099 cm^2^, number of pixels 42) is shown in red and the reference ROI in blue. **b** CSF flow velocity is shown for the different pixels with mean flow velocity of all pixels indicated by a dark line, including noise level of pixels in reference tissue (colored lines) with mean noise level indicated by dark stippled line. The velocities from each pixel were assessed using MATLAB, and expressed as centimeters per second. Positive values refer to cranial CSF flow direction and negative values caudal CSF flow direction. The CSF flow for the different pixels is also indicated in 2D demonstrating (**c**) upward (retrograde) flow and (**d**) downward (antegrade) flow. 3D presentations of CSF flow are presented showing (**e**) upward flow, (**f**) combined, and (**g**) downward flow. Cranio-cervical junction: (**h**) The ROI (1.963 cm^2^, number of pixels 636) is shown in red and the reference ROI in blue. **i** CSF flow velocity is shown for the different pixels with mean flow velocity of all pixels indicated by a dark line, including noise level of pixels in reference tissue (colored lines) with mean noise level indicated by dark stippled line. The CSF flow for the different pixels is also indicated in 2D demonstrating (**j**) upward (retrograde) flow and (**k**) downward (antegrade) flow. 3D presentations of CSF flow are presented showing (**l**) upward flow, (**m**) combined, and (**n**) downward flow

The cardiac-gated phase-contrast MRI was obtained prior to intrathecal contrast administration. Details about the methodology for analysis of phase-contrast MRI has previously been reported (see Fig. 2a–n) [20]. Regions of interest (ROIs) were manually placed along the outer border of cerebral aqueduct (Additional file 1: Fig. S1) and the cranio-cervical junction (Additional file 1: Fig. S2). The velocities from each pixel were assessed in MATLAB (Mathworks, Natick, USA) and converted to centimeters per second, considering noise level [20].

Navier–Stokes equations with the assumption of fluid flow perpendicular to the acquisition plane was applied to compute the peak-peak pressure gradient (dP), as previously described in more detail [21].

Volumetric flow rate $Q$ (ml/s) in the cerebral aqueduct or cranio-cervical junction was determined by computing the sum of each pixel velocity over one cycle multiplied with pixel size

$$Q\left(t\right)= \left(\sum_{i=1}^{n}{v}_{i }\right)\times dx\times dy.$$

The positive and negative contributions of the volumetric flow rate were calculated separately in the same manner, and the positive and negative velocities were extracted before the calculation. Thereby, net volume over one cycle was calculated by discrete integration (trapezoidal method) of $Q$ over time:

$$Net volume= \frac{dt}{2}\sum_{i=1}^{n}(Q({t}_{i+1})+Q({t}_{i })).$$

Volumes over one cycle in cranial and caudal direction were calculated by integration of positive and negative volume fluxes over time. The CSF volumetric net flow rate during one cycle (ml/cycle) was determined by the sum of the positive and negative CSF flux.

With regard to estimating CSF flow volume over 24 h, the daily CSF volumetric net flow rate, expressed in liter (L) over 24 h was estimated by multiplying the CSF net flow volume over one cardiac cycle with the heart rate (HR) and then multiplied with 1440 (min/day). The HR was determined over the MRI scan time that was 6 min.

Finally, we calculated the ratio between the retrograde CSF volumetric net flow rate per cardiac cycle at the cerebral aqueduct versus the CCJ cranial net flow rate per cycle, according to this formula (CSF_Aq-CCJ_-ratio):

$$\frac{{Vol}_{A}}{{Vol}_{CCJ}}\times 100,$$

where ${Vol}_{A}$ is the net retrograde volume of CSF flow during one cycle measured in the cerebral aqueduct and ${Vol}_{CCJ}$ is the net cranial-directed volume of CSF flow during one cycle measured in CCJ. Upward net CSF flow is considered to be an estimate of the portion of CSF that distributes from CCJ into the supra-aqueductal compartment during one cardiac cycle. It has previously been determined a ratio between average stroke volume at the aqueduct and CCJ levels though not considering net volumes [22, 23].

We estimated the noise level by calculating the signal-to-noise ratio (SNR), defined as

$$SNR=10\times log\left(\frac{{P}_{S}}{{P}_{N}}\right),$$

${P}_{S}$ is the power of the signal and ${P}_{N}$ is the power of the noise, where power is defined as the amplitude squared. The signal was defined as the mean velocity in the ROI, and we calculated SNR for each pixel in the reference ROI. To achieve one representative SNR for each data set, we averaged the calculated SNR numbers.

### Pulsatile ICP score indicative of intracranial compliance

Within the Department of neurosurgery, overnight monitoring of pulsatile ICP is utilized as a surrogate marker of the intracranial compliance, i.e., pressure–volume reserve capacity [24] and the static ICP (mean ICP) is also measured. The procedure has been described in detail before [24]. In short, an ICP sensor was placed in the brain parenchyma through a small burr hole in the skull under local anesthesia, and monitoring done over-night using a computerized system. The mean ICP wave amplitude (MWA) is the pressure difference between the systolic maximum and diastolic minimum pressures for cardiac-induced single ICP waves during consecutive 6-s time intervals. The static ICP (mean ICP) is the absolute pressure difference between the intracranial compartment and reference atmospheric pressure.

### Statistical analyses

The statistical analysis was performed using the SPSS software version 26 (IBM Corporation, Armonk, NY, USA). Continuous data are presented as mean with standard deviation, and categorical data as numbers and percentages. Difference between patient groups versus the REF cohort was determined by independent samples t-test, and differences between categorical data were determined by the Pearson Chi-square test. Pearson correlation coefficients were determined to examine associations between different variables. All observations for a specific variable were used when determining the correlation coefficients. Statistical significance was accepted at the 0.05 level (two-tailed).

## Results

### Patient cohorts

The study included 94 consecutive individuals who underwent a standardized MRI protocol, incorporating both intrathecal contrast enhanced MRI and phase-contrast MRI, during the period October 2015 to June 2018. Twenty-four individuals were categorized as reference (REF) patients where no apparent CSF disturbance was eventually diagnosed, and 70 patients with CSF disorders, including idiopathic normal pressure hydrocephalus (iNPH, n = 34), communicating hydrocephalus (cHC, n = 7), spontaneous intracranial hypotension (SIH, n = 8), arachnoid cyst (AC, n = 7), pineal cyst (PC, n = 11) and idiopathic intracranial hypertension (IIH, n = 3; Table 1). Compared to the REF group, age was higher in the iNPH and SIH groups, and gender distribution differed in the iNPH group. Ventricular size indices of CSF space anatomy were significantly increased in the iNPH, cHC and AC groups, and mean ICP wave amplitude (MWA), serving as a surrogate marker of intracranial compliance, was significantly increased (indicative of impaired intracranial compliance) in the iNPH, cHC and IIH patients (Table 1).

**Table 1:** Patient data regarding demography, CSF space anatomy, MRI acquisitions and ICP scores

		Total	REF	iNPH	cHC	SIH	AC	PC	IIH
Demographic									
N		94	24	34	7	8	7	11	3
Age (years)		50.4 ± 18.8	36.1 ± 10.6	70.8 ± 6.4^c^	37.4 ± 11.8	50.6 ± 16.2^b^	42.3 ± 14.7	35.8 ± 10.4	36.7 ± 14.2
Gender (f/m)		57/37	19/5	10/24^c^	4/3	6/2	5/2	10/1	3/0
Height (m)		1.73 ± 0.09	1.72 ± 0.08	1.75 ± 0.09	1.73 ± 0.09	1.68 ± 0.06	1.75 ± 0.10	1.70 ± 0.06	1.71 ± 0.13
Weight (kg)		81.0 ± 18.6	78.8 ± 15.8	84.0 ± 19.4	79.1 ± 28.1	76.0 ± 20.0	79.9 ± 12.4	80.5 ± 18.4	86.0 ± 26.2
BMI (kg/m^2^)		26.9 ± 5.0	26.5 ± 4.9	27.0 ± 4.3	25.9 ± 6.6	26.9 ± 7.0	26.0 ± 3.1	27.9 ± 5.9	29.0 ± 6.2
HR (beats/min)		68.4 ± 10.5	70.1 ± 12.3	68.9 ± 9.7	64.2 ± 17.3	66.3 ± 5.9	65.4 ± 7.1	68.3.9 ± 10.4	68.0 ± 7.8
CSF space anatomy									
Evans index		0.31 ± 0.07	0.26 ± 0.03	0.39 ± 0.03^c^	0.32 ± 0.03^c^	0.28 ± 0.03	0.27 ± 0.05	0.25 ± 0.03	0.28 ± 0.04
Callosal angel		99 ± 28	116 ± 11	70 ± 22^c^	120 ± 14	116 ± 11	108 ± 18	117 ± 13	103 ± 20
DESH									
Present		19 (20%)	0	19 (56%)^c^	0	0	0	0	0
Absent		75 (80%)	24 (100%)	15 (44%)	7 (100%)	8 (100%)	7 (100%)	11 (100%)	3 (100%)
Volume 4th ventricle (ml)		2.3 ± 1.7	1.4 ± 0.3	3.6 ± 2.2^c^	1.8 ± 0.8	1.7 ± 0.8	1.8 ± 0.7^a^	1.4 ± 0.6	1.8 ± 0.6
Volume 3rd ventricle (ml)		2.0 ± 1.5	0.9 ± 0.4	3.7 ± 1.2^c^	1.8 ± 0.9^b^	1.3 ± 0.7	1.6 ± 0.9^b^	0.9 ± 0.4	1.2 ± 0.8
Volume lateral ventricles (ml)		62.6 ± 61.7	18.7 ± 13.3	137.7 ± 43.2^c^	45.7 ± 27.5^b^	26.5 ± 15.1	26.8 ± 24.1	13.3 ± 7.1	34.8 ± 23.8
MRI acquisitions									
Intrathecal contrast-enhanced MRI (N)		88	23	30	7	8	6	11	3
Phase-contrast MRI Aq (N)		85	23	32	5	4	7	11	3
Phase-contrast MRI CCJ (N)		32	4	18	3	4	2	1	0
Overnight ICP									
Static ICP									
Mean ICP average (mmHg)		6.7 ± 5.7	9.4 ± 6.8	4.6 ± 5.0^a^	11.7 ± 6.1	3.9	–	8.0 ± 4.4	8.5 ± 4.6
Mean ICP > 15 mmHg (%)		11 ± 22	23 ± 39	5 ± 11^a^	33 ± 33	0	–	10 ± 17	7 ± 6
Pulsatile ICP									
MWA average (mmHg)		5.1 ± 1.9	3.2 ± 0.6	5.9 ± 1.8^c^	5.3 ± 2.1^a^	3.4	–	3.5 ± 0.8	5.5 ± 0.4^c^
MWA > 5 mmHg (%)		42 ± 34	4 ± 4	57 ± 31^c^	44 ± 32^b^	2	–	7 ± 6	56 ± 6^c^

*AC* arachnoid cyst, *Aq* aqueduct, *BMI* body mass index, *CCJ* cranio-cervical junction, *cHC* communicating hydrocephalus, *DESH* disproportionately enlarged subarachnoid space hydrocephalus, *HR* heart rate (derived from phase-contrast MRI), *ICP* intracranial pressure, *IIH* idiopathic intracranial hypertension, *iNPH* idiopathic normal pressure hydrocephalus, *MWA* mean wave amplitude, *PC* pineal cyst, *REF* reference cohort, *SIH* spontaneous intracranial hypotension

Differences from the REF group were determined by independent samples t-test for continuous variables and by Pearson Chi-square test for categorical variables (^a^P < 0.05, ^b^P < 0.01, ^c^P < 0.001)

### Tracer enrichment of cerebral ventricles

The spinal transfer time, i.e. the time from lumbar intrathecal administration of tracer until first appearance of tracer at the level of cisterna magna, was comparable between all patient groups, and was 17.2 ± 16.1 min in the total group (Table 2).

**Table 2:** Measures of CSF tracer movement from intrathecal contrast-enhanced MRI

		Total	REF	iNPH	cHC	SIH	AC	PC	IIH
Spinal transit time (min)		17.2 ± 16.1	14.4 ± 9.6	19.3 ± 14.2	34.1 ± 43.8	14.3 ± 4.4	10.0 ± 2.1	13.7 ± 4.9	13.7 ± 7.5
Qualitative measures (N)		92	23	34	7	7	7	11	3
Ventricular reflux N (%)									
Grade 0		25 (27)	13 (57)	0	1 (14)	3 (43)	1 (14)	6 (55)	1 (33)
Grade 1		9 (10)	3 (13)	2 (6)	0	1 (14)	0	1 (9)	2 (67)
Grade 2		12 (13)	5 (22)	0	1 (14)	0	3 (43)	3 (27)	0
Grade 3		21 (23)	2 (9)	11 (32)	3 (43)	3 (43)	1 (14)	1 (9)	0
Grade 4		25 (27)	0	21 (62)	2 (29)	0	2 (29)	0	0
Percentage change in tracer enrichment (N)		88	23	30	7	8	6	11	3
4th ventricle									
1 h		1076 ± 1253	403 ± 595	1606 ± 1483^b^	443 ± 516	1138 ± 1571	1993 ± 1174^c^	847 ± 953	1296 ± 1142^a^
2 h		1495 ± 1185	746 ± 602	2248 ± 1187^c^	1397 ± 844^a^	1526 ± 1449	2112 ± 1175^b^	799 ± 875	1061 ± 1407
4 h		1414 ± 1112	762 ± 617	2181 ± 1083^c^	1411 ± 871^a^	1112 ± 1266	1985 ± 1192^b^	847 ± 855	698 ± 1031
6 h		1497 ± 1077	799 ± 642	2318 ± 938^c^	1626 ± 747^a^	1221 ± 1139	1867 ± 1210^b^	844 ± 823	972 ± 1065
24 h		559 ± 609	169 ± 168	1169 ± 595^c^	537 ± 569^b^	130 ± 94	555 ± 354^b^	160 ± 134	102 ± 00
3rd ventricle									
1 h		430 ± 675	140 ± 379	678 ± 779^b^	217 ± 303	563 ± 868	1234 ± 857^c^	106 ± 191	62 ± 77
2 h		728 ± 885	271 ± 451	1099 ± 926^c^	870 ± 752^a^	739 ± 1031	1626 ± 1341^c^	160 ± 197	212 ± 257
4 h		859 ± 965	332 ± 516	1364 ± 958^c^	1158 ± 978^a^	760 ± 1034	1774 ± 1397^b^	198 ± 285	134 ± 151
6 h		960 ± 968	419 ± 548	1638 ± 916^c^	987 ± 903	724 ± 857	1680 ± 1357^b^	253 ± 314	160 ± 39
24 h		479 ± 584	86 ± 116	1081 ± 543^c^	469 ± 383^c^	103 ± 102	482 ± 575^b^	56 ± 73	36 ± 36
Lateral ventricles									
1 h		333 ± 795	119 ± 356	446 ± 1001	245 ± 496	525 ± 1080	1216 ± 1151^b^	49 ± 112	7 ± 27
2 h		850 ± 1332	231 ± 424	1352 ± 1554^b^	1047 ± 1300^a^	939 ± 1556	1939 ± 2046^b^	98 ± 199	245 ± 315
4 h		1246 ± 1650	393 ± 755	2188 ± 1818^c^	1725 ± 1821^a^	1018 ± 1583	2176 ± 2263^b^	144 ± 305	158 ± 196
6 h		1461 ± 1687	521 ± 802	2785 ± 1737^c^	1338 ± 1447	1019 ± 1327	2125 ± 2097^b^	229 ± 433	140 ± 17
24 h		832 ± 1119	92 ± 188	2038 ± 1062^c^	676 ± 615^c^	110 ± 114	609 ± 848^b^	47 ± 75	49 ± 59

*AC* arachnoid cyst, *cHC* communicating hydrocephalus, *IIH* idiopathic intracranial hypertension, *iNPH* idiopathic normal pressure hydrocephalus, *PC* pineal cyst, *REF* reference cohort, *SIH* spontaneous intracranial hypotension

Differences from the REF group were determined by independent samples t-test (^a^P < 0.05, ^b^P < 0.01, ^c^P < 0.001)

The qualitative determination of ventricular reflux grade was made in 92 patients (24 h MRI was lacking in two individuals), in whom a Reflux Grade of 3–4 was observed in 46/92 (50%) patients (Table 2). As compared with the REF cohort, the Ventricular reflux grade distribution was significantly different in the iNPH (p < 0.001), cHC (p = 0.009); and AC cohorts (p = 0.031; Pearson Chi-square test; Table 2).

The FreeSurfer based quantitative estimation of tracer enrichment within cerebral ventricles over time showed significantly stronger enrichment within third and lateral ventricles of the iNPH, cHC and AC patients, as compared to the REF cohort (Table 2). Significant tracer enrichment within the third and lateral ventricles after 1–2 h was seen in the iNPH, cHC and AC cohorts (Table 2).

**Fig. 3:**
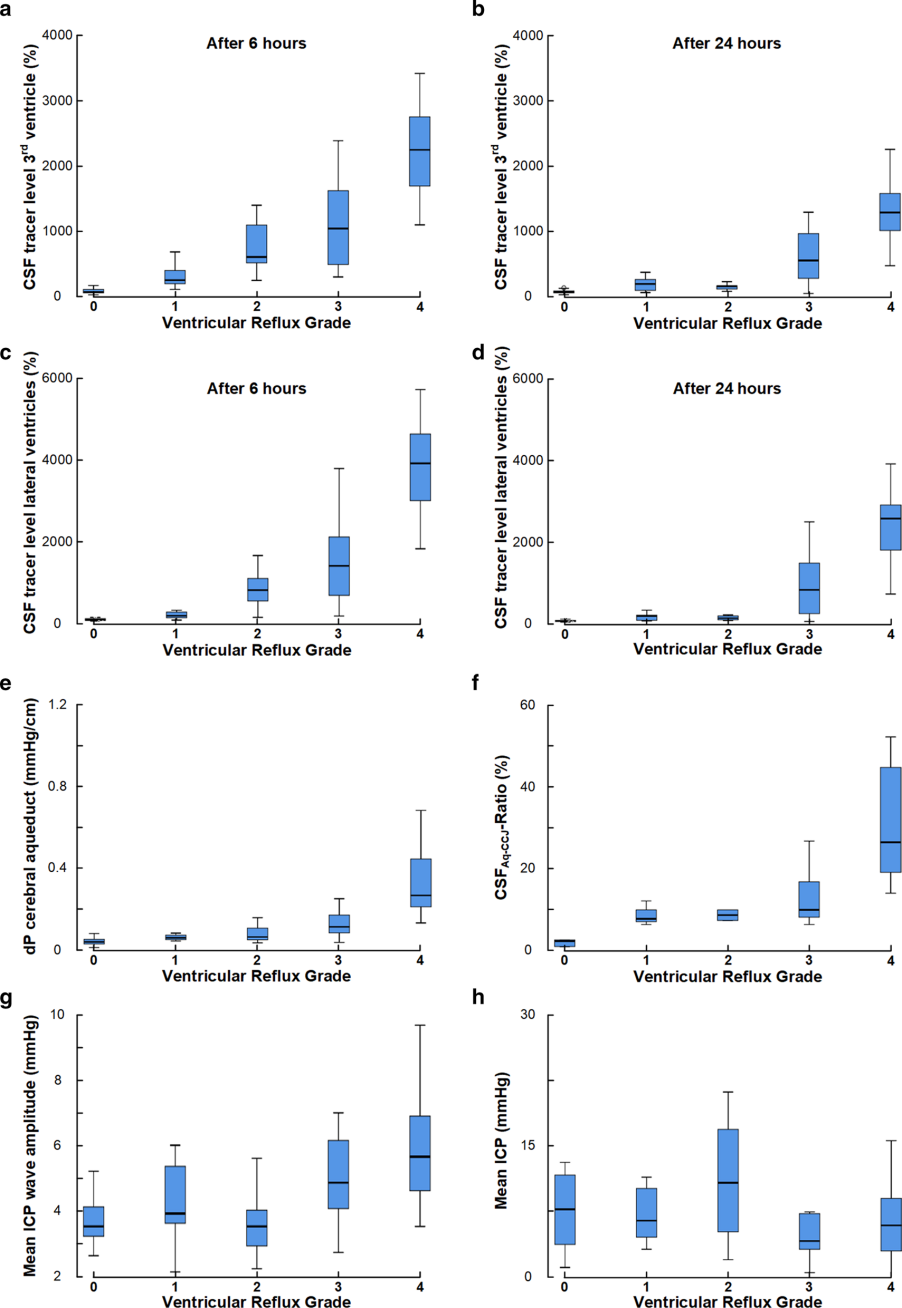
Ventricular reflux grades 0–4, describing degree of supra-aqueductal tracer enrichment, are associated with other independent measures of CSF tracer transport and CSF flow. For Ventricular Reflux Grades 0 to 4, there were significant differences in tracer enrichment in 3rd ventricle after 6 h (**a**; n = 79; P < 0.001), and 24 h (**b**; n = 86; P < 0.001), and differences in tracer enrichment within lateral ventricles after 6 h (**c**; n = 76; P < 0.001), and 24 h (**d**; n = 86; P < 0.001). The Ventricular Reflux Grades 0–4 also differentiated pressure-gradient (dP) in cerebral aqueduct (**e**; n = 83; P < 0.001), and the CSF_Aq-CCJ_-Ratio (**f**; n = 27; P < 0.001), i.e. a high degree of supra-aqueductal tracer enrichment was related to a higher pressure gradient in the cerebral aqueduct and also a higher proportion of cranial directed CSF flow in CCJ be transported retrograde in the cerebral aqueduct. The Ventricular Reflux Grade also differentiated mean ICP wave amplitude (MWA) serving as surrogate marker of intracranial compliance (**g**; n = 53; P = 0.001), though the static ICP (mean ICP) was not different between the Ventricular Reflux Grades (**h**; n = 51; P = 0.349). Hence, higher MWA, indicative of impaired intracranial compliance was associated with higher degree of supra-aqueductal CSF tracer enrichment. Data presented as box plots with 25th and 75th percentiles and ranges. Statistical differences determined by one-way ANOVA with Bonferroni-corrected post hoc tests

Considering the entire patient material, the ventricular reflux grades related to other and independent variables of CSF tracer movement and CSF flow that were measured in this study. There was a significant association between ventricular reflux grade and tracer enrichment in third ventricle after 6 h (Fig. 3a) and 24 h (Fig. 3b) and tracer enrichment in lateral ventricles after 6 h (Fig. 3c) and 24 h (Fig. 3d). Moreover, increasing ventricular reflux grade was accompanied with increased pressure gradient (dP) in cerebral aqueduct (Fig. 3e) and with increased CSF_AQ/CCJ_-Ratio (Fig. 3f), i.e. increased ventricular reflux grade was accompanied with a higher proportion of net cranial CSF flow in cranio-cervical junction passing retrograde via the cerebral aqueduct. Increased ventricular reflux grade was as well accompanied with increasing MWA scores (Fig. 3g), i.e. impaired intracranial compliance was associated with increased ventricular reflux grade. There was also a significant positive correlation between MWA and quantitative measures of tracer enrichment in third ventricles after 6 h (Pearson correlation 0.390, P = 0.007) and 24 h (Pearson correlation 0.363, P = 0.010), and between MWA and tracer enrichment in lateral ventricles after 6 h (Pearson correlation 0.406, P = 0.005) and 24 h (Pearson correlation 0.390, P = 0.005Suppl. Figure 3). On the other hand, the static ICP (mean ICP) was not different for the various ventricular reflux grades (Fig. 3h), and was not correlated positively with quantitative measures of tracer enrichment (data not shown). Taken together, increasing MWA scores, indicative of impaired intracranial compliance, were accompanied by an increasing degree of supra-aqueductal tracer enrichment.

With regard to traditional biomarkers of CSF space, it may be noted that callosal angle (Table 1) becomes narrower with increasing degree of ventricular tracer reflux. Hence, a negative correlation was found between callosal angle and tracer enrichment in 3rd ventricle after 6 h (Pearson correlation − 0.532, P < 0.001) and 24 h (Pearson correlation − 0.651, P < 0.001), and between callosal angle and tracer enrichment in lateral ventricles after 6 h (Pearson correlation − 0.576, P < 0.001) and 24 h (Pearson correlation − 0.691, P < 0.001; Additional file 1: Fig. S4). Moreover, callosal angle was reduced in cases with increasing ventricular reflux grade (P < 0.001, one-way ANOVA).

### Fluid flow in the cerebral aqueduct and cranio-cervical junction

Phase-contrast MRI of the cerebral aqueduct was available in 85 patients (Table 3). With regard to direction-independent flow in the cerebral aqueduct, we found in the iNPH patients greater pressure gradient (dP), and in both iNPH and AC patients significantly greater total flow per cycle. The estimated absolute net volume over 24 h was increased in the iNPH, SIH and AC cohorts, with respective aqueduct net volumes of 1.087 ± 1.226 L/24 h, 1.127 ± 1.659 L/24 h, and 1.650 ± 2.836 L/24 h. In the total cohort of 85 patients, the estimated absolute net volume over 24 h was 0.790 ± 1.217 L/24 h (Table 3).

**Table 3:** PC-MRI-derived CSF volumetric net flow rates and directions in cerebral aqueduct

	Total	REF	iNPH	cHC	SIH	AC	PC	IIH
Total (N)	85	23	32	5	4	7	11	3
Direction-independent flow								
dP (mmHg/cm)	0.079 ± 0.035	0.068 ± 0.029	0.097 ± 0.035^b^	0.064 ± 0.020	0.060 ± 0.063	0.091 ± 0.034	0.056 ± 0.020	0.072 ± 0.025
Total flow/cycle (ml)	0.145 ± 0.175	0.067 ± 0.057	0.269 ± 0.223^c^	0.077 ± 0.038	0.058 ± 0.054	0.140 ± 0.129^a^	0.041 ± 0.023	0.052 ± 0.013
Absolute net flow (ml/cycle)	0.008 ± 0.013	0.004 ± 0.002	0.011 ± 0.012^b^	0.003 ± 0.002	0.011 ± 0.017^a^	0.018 ± 0.031^a^	0.004 ± 0.004	0.007 ± 0.011
Estimated absolute net flow (L/24 h)	0.790 ± 1.217	0.382 ± 0.232	1.087 ± 1.226^b^	0.236 ± 0.143	1.127 ± 1.659^a^	1.650 ± 2.836^a^	0.330 ± 0.374	0.916 ± 1.117^a^
Antegrade-directed net CSF flow								
N (%)	59 (69%)	19 (83%)	18 (56%)^a^	2 (40%)^a^	3 (75%)	5 (71%)	10 (91%)	2 (67%)
Volume (ml/cycle)	0.007 ± 0.010	0.004 ± 0.002	0.012 ± 0.014^a^	0.003 ± 0.000	0.014 ± 0.019^a^	0.007 ± 0.005^a^	0.003 ± 0.002	0.001 ± 0.000
Estimated volume (L/24 h)	0.681 ± 0.971	0.417 ± 0.234	1.181 ± 1.432^a^	0.223 ± 0.029	1.408 ± 1.911^a^	0.698 ± 0.519	0.225 ± 0.149^a^	0.280 ± 0.261
Retrograde-directed net CSF flow								
N (%)	26 (31%)	4 (17%)	14 (44%)	3 (60%)	1 (25%)	2 (29%)	1 (9%)	1 (33%)
Volume (ml/cycle)	0.011 ± 0.018	0.002 ± 0.002	0.010 ± 0.010	0.002 ± 0.002	0.003	0.044 ± 0.061	0.013	0.020
Estimated volume (L/24 h)	1.039 ± 1.642	0.215 ± 0.146	0.966 ± 0.935	0.245 ± 0.201	0.283	4.031 ± 5.595	1.374	2.189
Other variables								
ROI (cm^2^)	0.049 ± 0.039	0.028 ± 0.010	0.083 ± 0.043^c^	0.034 ± 0.015	0.029 ± 0.012	0.041 ± 0.022^a^	0.021 ± 0.007^a^	0.028 ± 0.010
SNR	19.894 ± 3.700	19.145 ± 4.144	20.691 ± 2.573	20.484 ± 2.189	16.739 ± 6.800	22.080 ± 4.692	19.088 ± 3.900	18.225 ± 0.701
Min velocity (cm/s)	-5.273 ± 2.437	-4.455 ± 1.892	-6.465 ± 2.468^b^	-4.623 ± 1.667	-5.140 ± 5.284	-6.209 ± 1.533^a^	-3.783 ± 1.335	-3.381 ± 0.403
Max velocity (cm/s)	4.075 ± 1.840	3.689 ± 1.923	4.771 ± 1.594^a^	3.522 ± 1.096	2.291 ± 1.468	4.828 ± 2.616	3.309 ± 1.289	3.963 ± 2.422

*AC* arachnoid cyst, *cHC* communicating hydrocephalus, *dP* pressure gradient, *IIH* idiopathic intracranial hypertension, *iNPH* idiopathic normal pressure hydrocephalus, *Max* maximum, *Min* minimum, *PC* pineal cyst, *REF* reference cohort, *ROI* region of interest, *SIH* spontaneous intracranial hypotension, *SNR* signal to noise ratio

Differences from the REF group were determined by independent samples t-test for continuous variables and Pearson Chi square test for categorical data (^a^P < 0.05, ^b^P < 0.01, ^c^P < 0.001)

With regard to ventricular reflux grades, total aqueductal flow per cycle was significantly higher in individuals with ventricular reflux grade 4 than grades 0 to 3 (P < 0.001) and absolute net flow per cycle was increased with reflux grade 4 as compared with grades 0–2 (P < 0.05; one-way ANOVA with Bonferroni-corrected posthoc test). Moreover, we found a significant positive correlation between magnitudes of total aqueductal flow and enrichment of tracer in 3rd ventricle at 6 h (Pearson correlation 0.611, P < 0.001) and 24 h (Pearson correlation 0.629, P < 0.001), and a significant positive correlation between total aqueductal flow and enrichment of tracer in lateral ventricle at 6 h (Pearson correlation 0.654, P < 0.001) and 24 h (Pearson correlation 0.625, P < 0.001; Additional file 1: S5).

Among the 85 individuals with phase-contrast MRI of the cerebral aqueduct, we found in 1/3 patients evidence of retrograde aqueduct flow, i.e. net flow from 4th to 3rd ventricle, and an antegrade net flow direction in 2/3 patients. Compared with REF patients, there was a higher proportion of retrograde net flow in iNPH (p = 0.040) and cHC patients (p = 0.046; Pearson Chi-square test; Table 3). It may be noted that the estimated retrograde net flow over 24 h was 0.966 ± 0.935 L/24 h in the 14/32 iNPH patients with retrograde aqueduct flow, and 4.031 ± 5.595 L/24 h in the 2/7 AC patients with retrograde aqueduct flow (Table 3). Moreover, estimated antegrade net flow over 24 h was 1.181 ± 1432 L/24 h in 18/32 iNPH patients with antegrade aqueduct flow, and 1.408 ± 1.911 L/24 h in 3/4 SIH patients with antegrade aqueduct flow. Accordingly, direction and magnitude of aqueduct flow vary substantially between patient cohorts with different CSF disorders.

Phase-contrast MRI of the cranio-cervical junction was available in 32 of the 94 individuals (Table 4). The pressure gradient (dP) and total flow per cycle in cranio-cervical junction were comparable across patient groups. Notably, net CSF flow was cranially directed in 25/32 (78%) individuals, with an estimated cranial-directed flow volume per day in these 25 patients of 4.736 ± 5.007 L/24 h. Conversely, among the about 7/32 (22%) individuals with spinally directed CSF flow in the cranio-cervical junction; the estimated volume per day was 4.688 ± 5.363 L/24 h (Table 4). The REF group included four individuals with phase-contrast MRI of the cranio-cervical junction. Net flow was cranial in all these four patients, with a flow volume per day 7.752 ± 10.615 L/24 h, whereas this was significantly lower in the iNPH cohort (Table 4). With regard to net flow in cranio-cervical junction, none of the patient groups differed from the REF cohort, which may be related to a low number of individuals in each group. The important findings were that net flow in cranio-cervical junction was in cranial direction in 4/5 patients and with high estimated volumes per day.

**Table 4:** PC-MRI-derived CSF volumetric net flow rates and directions in cranio-cervical junction

	Total	REF	iNPH	cHC	SIH	AC	PC	IIH
Total (CCJ; N)	32	4	18	3	4	2	1	0
Direction-independent flow								
dP (mmHg/cm)	0.040 ± 0.014	0.050 ± 0.012	0.038 ± 0.014	0.039 ± 0.012	0.042 ± 0.015	0.030 ± 0.000	0.067	–
Total flow/cycle (ml)	1.146 ± 0.452	1.796 ± 0.516	0.916 ± 0.358^b^	1.221 ± 0.201	1.239 ± 0.315	1.414 ± 0.059	1.537	–
Absolute net flow (ml/cycle)	0.045 ± 0.047	0.074 ± 0.098	0.037 ± 0.030	0.094 ± 0.049	0.015 ± 0.014	0.029 ± 0.005	0.082	–
Estimated absolute net flow (L/24 h)	4.726 ± 4.998	7.752 ± 10.615	4.118 ± 3.818^b^	8.371 ± 4.208	1.879 ± 2.198	2.540 ± 0.204	8.381	–
Cranially-directed net CSF flow								
N	25 (78%)	4 (100%)	14 (78%)	3 (100%)	2 (50%)	1 (50%)	1 (100%)	–
Volume (ml/cycle)	0.047 ± 0.049	0.074 ± 0.098	0.033 ± 0.024	0.094 ± 0.049	0.009 ± 0.001	0.032	0.082	–
Estimated volume (L/24 h)	4.736 ± 5.007	7.752 ± 10.615	3.529 ± 2.557	8.371 ± 4.208	0.911 ± 0.150	2.684	8.381	–
Spinally-directed net CSF flow								
N	7 (22%)	0	4 (22%)	0	2 (50%)	1 (50%)	0	0
Volume (ml/cycle)	0.039 ± 0.038	–	0.051 ± 0.047	–	0.021 ± 0.022	0.026	–	–
Estimated volume (L/24 h)	4.688 ± 5.363	–	6.182 ± 6.853	–	2.848 ± 3.275	2.396	–	–
CSF_Aq-CCJ_-ratio (%)	15.5 ± 14.1	2.4 ± 2.6	21.9 ± 14.1^b^	7.9 ± 2.0^a^	3.0 ± 3.6	24.9 ± 16.4^a^	6.6	–
Other variables								
ROI (cm^2^)	1.671 ± 0.495	1.994 ± 0.657	1.585 ± 0.458	1.497 ± 0.466	1.766 ± 0.578	2.111 ± 0.036	1.191	–
SNR	13.205 ± 6.263	16.745 ± 6.043	10.174 ± 4.285^a^	15.863 ± 5.553	20.295 ± 5.592	8.845 ± 0.259	25.975	–
Min velocity (cm/s)	− 1.832 ± 0.784	− 2.500 ± 0.683	− 1.615 ± 0.835	− 2.089 ± 0.419	− 1.892 ± 0.696	− 1.478 ± 0.022	− 2.778	–
Max velocity (cm/s)	1.011 ± 0.420	1.335 ± 0.413	0.861 ± 0.334^a^	1.200 ± 0.508	1.178 ± 0.555	0.766 ± 0.065	1.678	–

*AC* arachnoid cyst, *Aq* Aqueduct, *CCJ* cranio-cervical junction, *cHC* communicating hydrocephalus, *dP* pressure gradient, *IIH* idiopathic intracranial hypertension, *iNPH* idiopathic normal pressure hydrocephalus, *Max* maximum, *Min* minimum, *PC* pineal cyst, *REF* reference cohort, *ROI* region of interest, *SIH* spontaneous intracranial hypotension, *SNR* signal to noise ratio

Differences from the REF group were determined by independent samples t-test (^a^P < 0.05, ^b^P < 0.01, ^c^P < 0.001)

There was no significant correlation between magnitudes of total and net flow in the cerebral aqueduct (Pearson correlation − 0.068, P = 0.53) or the cranio-cervical junction (Pearson correlation 0.196, P = 0.282; Additional file 1: S6).

The CSF_Aq-CCJ_-Ratio represents the percentage of cranial-directed net flow in the cranio-cervical junction transferred retrograde via the cerebral aqueduct to supra-aqueductal ventricles. An increased ratio thus indicates ventricular redistribution of CSF flow, and this ratio was significantly higher in the iNPH, cHC and AC cohorts (Table 4). The CSF_Aq-CCJ_-Ratio correlated positively with enrichment of 3^rd^ ventricle after 6 (Pearson correlation 0.459, P = 0.018) and 24 h (Pearson correlation 0.469, P = 0.012), and with enrichment of lateral ventricles after 6 h (Pearson correlation 0.520, P = 0.006) and 24 h (Pearson correlation 0.501, P = 0.007; Additional file 1: Fig. S7). There also was a positive correlation between the CSF_Aq-CCJ_-Ratio and the mean ICP wave amplitude (Pearson correlation 0.512, P = 0.030), but not with mean ICP (Pearson correlation − 0.086, P = 0.733; Chi-square test; Additional file 1: Fig. S8ab). We also noted a significant positive correlation between the CSF_Aq-CCJ_-Ratio and the aqueductal pressure gradient (dP) (Pearson correlation 0.76, P < 0.001; Additional file 1: Fig. S8c), perhaps indicating that the pressure gradient is directed towards the third ventricle. On the other hand, the pressure gradient of the cranio-cervical junction was not significantly correlated with the CSF_Aq-CCJ_-Ratio (Additional file 1: Fig. S8d).

### Phase-contrast MRI-derived pressure gradients

**Fig. 4:**
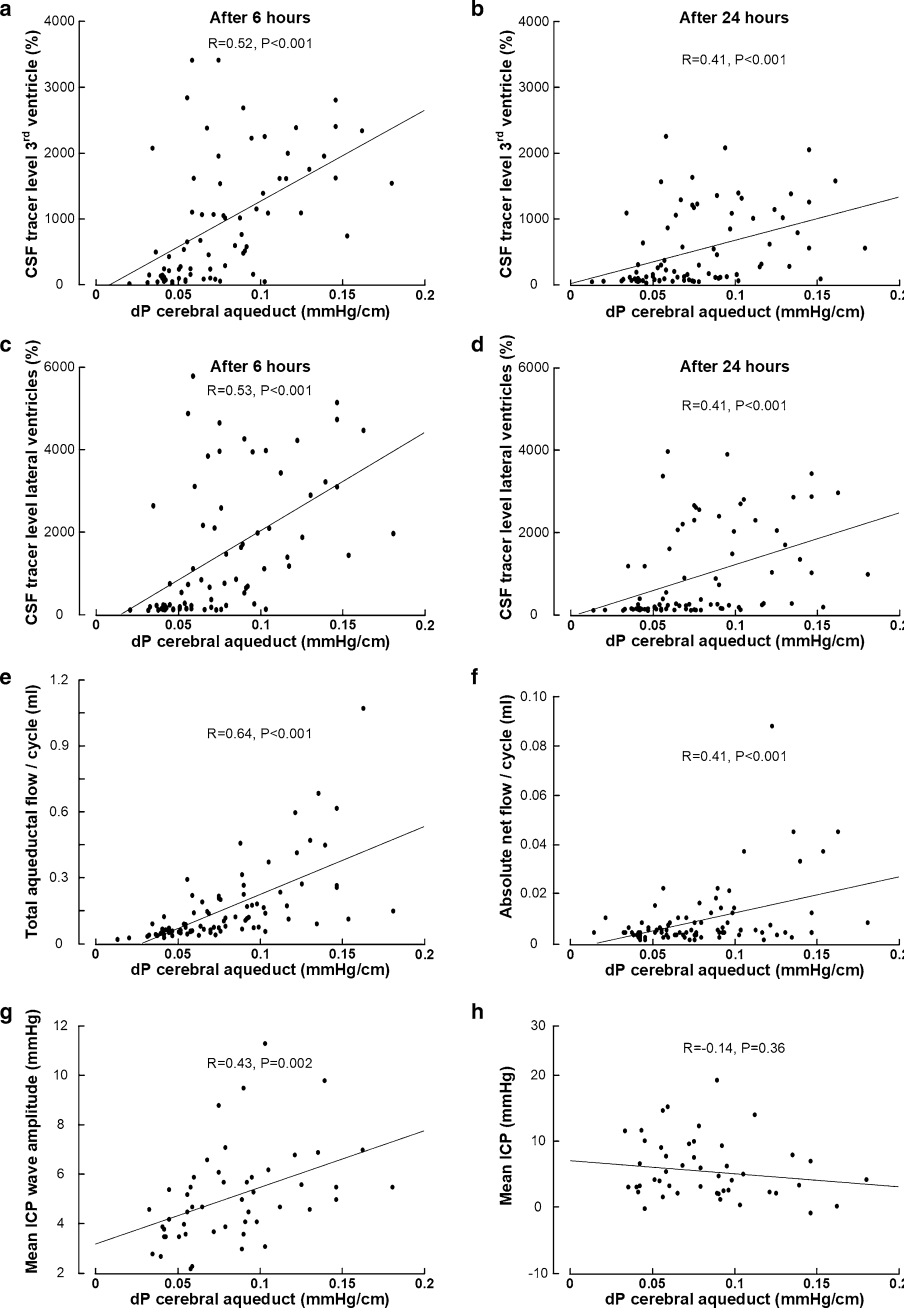
The pressure-gradient within the cerebral aqueduct correlates with several independent measures of CSF tracer movement and CSF flow. There was a significant positive correlation between the pressure-gradient (dP) and tracer enrichment within 3rd ventricle after 6 h (**a**; n = 74) and 24 h (**b**; n = 80), and tracer enrichment within lateral ventricles after 6 h (**c**; n = 74) and 24 h (**d**; n = 80). The pressure gradient (dP) also correlated positively with the total flow per cycle in the cerebral aqueduct (**e**; n = 85), the absolute net flow per cycle in the cerebral aqueduct (**f**; n = 85), and with the average overnight mean ICP wave amplitude indicative of the intracranial compliance (**g**; n = 50), whereas no correlated was found with static ICP (mean ICP, **h**; n = 48). Each plot shows the fit line with Pearson correlation coefficients and significance levels

The phase-contrast MRI-derived aquedutal pressure gradient (dP) is non-directional, i.e. the direction of the gradient cannot be determined from the present observations. An increasing pressure gradient (dP) was accompanied with more abnormal measures of the other independent variables of ventricular tracer transport and CSF flow. We found a positive correlation between the pressure-gradient (dP) and the degree of tracer enrichment in the 3rd ventricle (Fig. 4a–b) and lateral ventricle (Fig. 4c–d). There also was a positive correlation between the pressure gradient (dP) and the total aqueductal flow per cycle (Fig. 4e) and with the absolute net flow per cycle (Fig. 4f). We also found a significant positive correlation between pressure gradient (dP) and the mean ICP wave amplitude (MWA), indicative of the intracranial compliance (Fig. 4g). On the other hand, there was no correlation between dP and the static ICP (mean ICP) (Fig. 4h).

The aqueductal pressure gradient (dP) also was associated with the MRI biomarkers of CSF space anatomy, namely Evans’ index (Pearson correlation 0.508, P < 0.001) and callosal angle (Pearson correlation -0.446, P < 0.001; Additional file 1: Fig. S9).

The pressure gradient (dP) of the cranio-cervical junction was not associated with supra-aqueductal ventricular tracer enrichment, ventricular reflux grade, ICP scores (MWA or mean ICP), or MRI biomarkers of CSF space anatomy (Evans’ index and callosal angle) (data not shown).

## Discussion

The present observations disclosed marked variation in direction and magnitude of CSF flow within and between patient groups with evidence of re-direction of CSF flow towards ventricles in individuals with communicating hydrocephalus. The phase-contrast MRI data further suggest a significant extra-cranial CSF contribution to the water component of CSF.

We addressed neurological diseases characterized by different types of disturbed CSF homeostasis. Obstruction to CSF efflux is considered a main mechanism behind e.g. idiopathic normal pressure hydrocephalus (iNPH) and other communicating hydrocephalus (cHC) conditions, and is the rationale behind CSF diversion surgery (shunt surgery) [25]. In spontaneous intracranial hypotension (SIH), on the other hand, abnormal high CSF efflux is the tentative mechanism, usually caused by CSF leakage that may require intervention to prevent further leakage [26]. Brain cysts such as arachnoid cysts (ACs) and pineal cysts (PCs) may alter CSF flow in subarachnoid spaces due to local mass effect [27], and sometimes require cyst removal to ameliorate the CSF obstruction. Idiopathic intracranial hypertension (IIH) may be treated with the carbonic acid dehydrase inhibitor acetazolamide, which reduces CSF production [28]. In IIH, over-production of CSF is one tentative mechanism.

As CSF tracer, we used the MRI contrast agent gadobutrol, which is a hydrophilic molecule of about 604 Da [15]. It should be noted that even though long-term tracer movement derives from CSF flow, they are not synonymous. Furthermore, the MRI T1 sequences do not allow for determination of absolute quantities of tracer. Nevertheless, the tracer movement may be considered a surrogate marker of CSF flow, as the hydrophilic CSF tracer would be expected to follow the route of water movement and thereby provide an indirect measure of molecular motions in CSF over time. Using this methodology, we have previously shown that a CSF tracer freely communicates with interstitial fluid of the entire brain [15], and potentially directly with the meningeal lymphatic structures through the parasagittal dura [29].

Since MRI based quantitative estimation of tracer enrichment would require acquisition of T1 maps, and CSF flow at phase-contrast MRI requires sophisticated post-processing, not easily performed in daily clinical practice, we recently introduced the qualitative grading of ventricular tracer reflux, based on visual inspection of intrathecal contrast enhanced MRI acquisitions [13]. Ventricular reflux grades 3–4 were found to characterize shunt-responsive iNPH (i.e., Definite iNPH according to the Japanese guidelines), and was proposed as MRI biomarker for shunt-responsive iNPH [13]. The present data extended previous observations that other types of communicating hydrocephalus than iNPH also present with ventricular reflux grades 3–4. Less than 10% of the present REF individuals without apparent CSF disturbance or patients with other CSF disturbances than communicating hydrocephalus presented with ventricular reflux grades 3–4. Of note is that the ventricular reflux grading compared well with the FreeSurfer-based quantification of tracer transport to the third and lateral ventricles, and compared with other independent MRI-based biomarkers of CSF flow, such as total aqueductal CSF flow and aqueductal pressure-gradient.

We also found that higher reflux grades corresponded to higher aqueductal pressure-gradient (dP) as well as increased overnight scores of the pulsatile ICP score mean ICP wave amplitude (MWA), the latter constituting a surrogate marker of impaired intracranial compliance, i.e. impaired intracranial pressure–volume reserve capacity [30]. This finding further underpins a role of impaired intracranial compliance in some CSF disorders.

Since iNPH patients were older than REF patients, the ventricular reflux grades 3–4 in iNPH could potentially be attributed to age alone. On the other hand, the cohort with communicating hydrocephalus was considerably younger and comparable with the references and those with obstruction of subarachnoid CSF, indicating that high degree of reflux in communicating hydrocephalus is not merely an age-related phenomenon. Further studies are required to examine whether degree of ventricular reflux of tracer tends to increase with age.

The magnitude and direction of CSF flow in the cerebral aqueduct and the cranio-cervical junction vary substantially within and between patients groups. We found antegrade net CSF flow within the cerebral aqueduct in the majority of REF patients (87%), while a higher proportion of retrograde net CSF flow was seen in some CNS diseases, such as communicating hydrocephalus. Among the 85 patients with aqueductal flow measurements, one third of patients presented with net retrograde aqueductal flow, with average net retrograde CSF flow volumes 1.039 ± 1.642 L/24 h. We previously reported retrograde aqueductal flow in 14/21 iNPH patients (estimated average net retrograde flow 1.1 ± 2.2 L/24 h) [20], and in 5/8 individuals with a previous subarachnoid hemorrhage (estimated average net retrograde flow 2.2 ± 3.7 L/24 h) [31]. It should further be noted that in REF patients, patients with a brain cyst (AC or PC), and patients with SIH, CSF flow was antegrade in the cerebral aqueduct with an estimated net volume 0.2–0.5 L/24 h, which compares well with the Third circulation concept.

A methodological strength with our approach of determining flow velocities is that CSF flow was calculated pixel-by-pixel level, i.e. flow velocity was determined for each individual pixel (1 × 1 × 1 mm^3^) and the average calculated from all pixels within the region of interest. Moreover, we corrected for background velocity offset, which may be machine-dependent [32]. The common approach in phase-contrast studies is to determine average flow velocity from all pixels of the defined region of interest, not analyzing each pixel separately. The traditional approach has a greater risk of including border zone pixel with partial volume effects than our pixel-based method.

Multiple groups have previously reported net retrograde CSF flow within the cerebral aqueduct of patients with communicating hydrocephalus [22, 33–37]. However, the exact volume estimates of net aqueductal flow are debated due to methodological limitations with the phase-contrast MRI technique. The present observations of supra-aqueductal tracer enrichment further support the existence of net retrograde aqueductal CSF flow. Reflux grades 3 to 4 were observed in 46/92 patients (50%), characterized by a high degree of tracer enrichment in lateral ventricles at 24 h. If the CSF of the ventricles is replaced by CSF reproduction three times over 24 h, the continued presence of tracer at 24 h suggests either reduced formation of CSF or increased dispersion. A recent nuclear medicine cisternogram study found the radiotracer to more frequently enrich in lateral ventricles, though being absent or delayed over cerebral convexity, of individuals with DESH sign and high convexity tight sulci [38]. The latter is seen in iNPH patients (see Table 1). It is also seen in 6.6% of general population above 50 years of age [39], indicating that CSF disturbance becomes more common with increasing age.

Even though the CSF production from choroid plexus has traditionally been estimated to about 80% [4], it is open to debate whether a significant contribution to the water component of CSF derives from extra-choroidal and even extra-cranial sources. The present observations suggest production of water at the level of the spinal canal. Within the cranio-cervical junction, 78% patients had cranial directed net CSF flow, and estimated net cranial CSF flow volume was 4.736 ± 5.007 L/24 h. In a previous study utilizing cardiac-gated phase-contrast MRI, we estimated volumetric net flow in cranial direction, providing evidence for spinal cord CSF production in four healthy individuals (estimated 6.4 ± 4.9 L/24 h) and in 26 iNPH patients (estimated 6.9 ± 9.0 L/24 h) [20]. Furthermore, in the present cohort of 94 individuals, average time from lumbar injection of tracer until first appearance in cranio-cervical junction was 17.2 ± 16.1 min. The underlying mechanisms need to be clarified, but this short spinal transit time may be attributed to a potent dispersion effect within the spinal canal [40].

These observations also add to the experimental evidence suggesting that spinal cord formation of water adds to the CSF in supine subjects. Animal studies show that the water channel aquaporin-4 (AQP4), mediating a significant net extra-choroidal formation of CSF water, is abundant in brain and particularly the spinal cord tissue [41], where perivascular spaces communicate directly with subarachnoid CSF [42, 43]. High rates of CSF secretion was previously shown from spinal cord ependyma [44], and a more recent study showed perivascular outflow of CSF from the entire spinal cord [42]. Since the CSF hydrostatic pressure strongly depends on body position [45], it may be assumed that posture is a major determinant for spinal cord CSF production. Our estimates are retrieved from individuals lying flat in the MRI machine.

We would like to stress that the presently reported volumes of CSF flow in cerebral aqueduct and cranio-cervical junction are derived from *cardiac-gated* phase-contrast MRI during daytime in wake individuals lying flat. The cardiac-gated phase-contrast MRI estimates net flow induced by the heartbeat. These volumes are, however, not synonymous with the total CSF production. It is well established that cardiac gated phase-contrast MRI does not incorporate CSF movements induced by respiration, which contributes significantly [46]. In a modeling study, we found the cardiac derived component of CSF displacement to be minor, as compared to the respiratory contribution [47]. In humans, forced inspiration produces cranially directed CSF flow in the entire fluid compartment from the lumbar to the cerebral aqueductal levels, whereas forced expiration has opposite effects (except for aqueductal flow) [48, 49]. In addition to the respiratory influence, low frequency vasomotor cycles may possibly affect CSF flow even more, though this latter phenomenon is presently less explored. Other factors are also at play. Day-night cycle may affect CSF production; a fourfold increase in CSF production was estimated during night, as compared to day-time [50]. Age and gender seems as well to affect CSF flow pattern [51]. The volumes of CSF flow probably differ extensively from the traditionally estimated CSF production rate of 0.5 L/24 h. In this regard, it is of particular interest that Bering in 1952 [52], based on studies with heavy water (D_2_O) in humans and animals, reported free and constant exchange of water between blood, brain and CSF. He gave evidence for a CSF formation rate > 22 ml/min, i.e. > 70 times more than an estimate production rate of 0.3 ml/min.

Some limitations should be noted. First, intrathecal gadobutrol is given off-label, which represents one important limitation with this method. We have, however, good experience regarding the safety of intrathecal gadobutrol in a dose of 0.5 mmol [53, 54]. Moreover, a recent systematic review including 1036 patients from 53 studies concluded that no serious adverse events have been reported when intrathecal gadolinium based contrast agents are given in a dose of 1.0 mmol or lower [55]. Our recent experience also shows that the diagnostic information of intrathecal gadobutrol is maintained at a dose of 0.25 mmol. Possibly, the dose may be lowered further. The clinical introduction of intrathecal gadobutrol would depend on the therapeutic index, i.e. the balance between safety concerns and clinical usefulness. Since the hydrophilic properties are much the same for gadobutrol and radiopaque contrast agents used on label intrathecally for CT cisternography, the method of assessing ventricular reflux of tracer may potentially have broader use, though limitations regarding radiation dose at CT would remain.

One limitation with the phase-contrast MRI methodology is the accuracy of calculation of very small volumes, which is related to variability of physiological measures such as heart rate. While retrospective cardiac gating has been shown more accurate than prospective cardiac gating as it enables continuous measurements throughout the cardiac cycle [56], the flow velocity curve is no real-time measurement, but averages measures over many cardiac cycles (usually over about 5–6 min). The averaging of flow velocity curves over several cardiac cycles introduces some smoothing of the flow velocity signal, thereby introducing some inaccuracy.

Another limitation should be noted regarding our estimation of CSF flow volumes over 24 h from measurements obtained over a few minutes. Obviously, these estimates are subject to uncertainty. On the other hand, it would be expected that physiology is rather stable.

## Conclusions

The direction and magnitude of CSF flow in cerebral aqueduct and cranio-cervical junction vary considerably across disease categories, with re-direction of CSF flow towards ventricles characterizing communicating hydrocephalus. Our observations add to the increasing body of evidence that the traditional concept of CSF homeostasis, represented by the third circulation, represents an over-simplification. A significant part of the CSF water component may be produced extra-cranially. While quantification of CSF flow by using phase-contrast MRI or CSF tracer enrichment require sophisticate post-processing, a qualitative assessment of ventricular tracer reflux grade may have better utility in clinical practice.

## Supplementary Information

**Additional** **file** **1:** Figure S1: Examples of phase-contrast MRI of the cerebral aqueduct. For different patients, the regions of interest within the CSF of the cerebral aqueduct is shown in red and the reference regions of interest in blue (a, c, e, g). The CSF flow velocity is shown for the different pixels with mean flow velocity of all pixels indicated by a dark line, including noise level of pixels in reference tissue (colored lines) with mean noise level indicated by dark stippled line (b, d, f, h). The velocities from each pixel were assessed using MATLAB, and expressed as centimeters per second. Positive values refer to cranial CSF flow direction and negative values caudal CSF flow direction. **Figure** **S2:** Examples of phase-contrast MRI of the cranio-cervical junction. For different patients, the regions of interest within the CSF of the cranio-cervical junction is shown in red and the reference regions of interest in blue (a, c). The CSF flow velocity is shown for the different pixels with mean flow velocity of all pixels indicated by a dark line, including noise level of pixels in reference tissue (colored lines) with mean noise level indicated by dark stippled line (b, d). The velocities from each pixel were assessed using MATLAB, and expressed as centimeters per second. Positive values refer to cranial CSF flow direction and negative values caudal CSF flow direction. **Figure** **S3:** Association between the mean ICP wave amplitude (MWA) and enrichment of tracer within third and lateral ventricles. There was a significant positive correlation between the mean ICP wave amplitude (MWA) measured over-night and tracer enrichment within 3rd ventricle after 6 hours (a; n=47) and 24 hours (b; n=50), and between MWA and tracer enrichment within lateral ventricles after 6 hours (c; n=47) and 24 hours (d; n=50). Each plot shows the fit line with Pearson correlation coefficients and significance levels. **Figure** **S4:** Association between the callosal angle and enrichment of tracer within third and lateral ventricles. There was a significant negative correlation between the callosal angle and tracer enrichment within 3rd ventricle after 6 hours (a; n=81) and 24 hours (b; n=88), and between callosal angle and tracer enrichment within lateral ventricles after 6 hours (c; n=81) and 24 hours (d; n=88). Each plot shows the fit line with Pearson correlation coefficients and significance levels. **Figure** **S5:** Association between the total aqueductal flow per cycle and enrichment of tracer within third and lateral ventricles. There was a significant positive correlation between the total aqueductal flow per cycle and tracer enrichment within 3rd ventricle after 6 hours (a; n=74) and 24 hours (b; n=80), and between total aqueductal flow per cycle and tracer enrichment within lateral ventricles after 6 hours (c; n=74) and 24 hours (d; n=80). Each plot shows the fit line with Pearson correlation coefficients and significance levels. **Figure** **S6:** Association between the total flow and net flow per cycle in cerebral aqueduct and craniocervical junction. There was no correlation between the total flow and net flow per cycle in (a) the cerebral aqueduct (n=85) and (b) the cranio-cervical junction (n=32). Each plot shows the fit line with Pearson correlation coefficients and significance levels. **Figure** **S7:** Association between the CSFAq-CCJ-Ratio and enrichment of tracer within third and lateral ventricles. There was a significant positive correlation between the CSFAq-CCJ-Ratio and tracer enrichment within 3rd ventricle after 6 hours (a; n=26) and 24 hours (b; n=28), and between the CSFAq-CCJ-Ratio and tracer enrichment within lateral ventricles after 6 hours (c; n=26) and 24 hours (d; n=28). Each plot shows the fit line with Pearson correlation coefficients and significance levels. **Figure** **S8:** Association between the CSFAq-CCJ-Ratio and the mean ICP wave amplitude (MWA and mean ICP, and between the CSFAq-CCJ-Ratio and the pressure gradient in the cerebral aqueduct and cranio-cervical junction. There was a significant positive correlation between the CSFAq-CCJ-Ratio and the mean ICP wave amplitude (a; n=18) but no correlation with the mean ICP (b; n=18). The CSFAq-CCJ-Ratio was positively correlated with the pressure gradient (dP) at the cerebral aqueduct (c; n=29), but not with the pressure gradient (dP) in the craniocervical junction (d; n=29). Each plot shows the fit line with Pearson correlation coefficients and significance levels. **Figure** **S9:** Association between the pressure gradient in the cerebral aqueduct and the biomarkers of CSF space Evan’s index and callosal angle. There was a significant positive correlation between the pressure gradient (dP) at the cerebral aqueduct and the Evan’s index (a; n=85), and a significant negative correlation between the pressure gradient (dP) at the cerebral aqueduct and the callosal angle (b; n=85). Each plot shows the fit line with Pearson correlation coefficients and significance levels.

## Abbreviations

AC: Arachnoid cyst
AQP4: Aquaporin-4
CCJ: Cranio-cervical junction
cHC: Communicating hydrocephalus
CNS: Central nervous system
CSF: Cerebrospinal fluid
DESH: Disproportionately enlarged subarachnoid space hydrocephalus
D_2_O: Heavy water
dP: Peak–peak pressure gradient
ICP: Intracranial pressure
GFR: Glomerular filtration rate
HR: Heart rate
IIH: Idiopathic intracranial hypertension
iNPH: Idiopathic normal pressure hydrocephalus
MRI: Magnetic resonance imaging
MWA: Mean wave amplitude
PC: Pineal cyst
REF: Reference
REK: Regional Committee for Medical and Health Research Ethics
ROI: Regions of interest
SIH: Spontaneous intracranial hypotension

## References

[1] Iliff JJ, Wang M, Liao Y, Plogg BA, Peng W, Gundersen GA, Benveniste H, Vates GE, Deane R, Goldman SA (2012). A paravascular pathway facilitates CSF flow through the brain parenchyma and the clearance of interstitial solutes, including amyloid beta. Sci Transl Med.

[2] Louveau A, Smirnov I, Keyes TJ, Eccles JD, Rouhani SJ, Peske JD, Derecki NC, Castle D, Mandell JW, Lee KS (2015). Structural and functional features of central nervous system lymphatic vessels. Nature.

[3] Aspelund A, Antila S, Proulx ST, Karlsen TV, Karaman S, Detmar M, Wiig H, Alitalo K (2015). A dural lymphatic vascular system that drains brain interstitial fluid and macromolecules. J Exp Med.

[4] Spector R, Robert Snodgrass S, Johanson CE (2015). A balanced view of the cerebrospinal fluid composition and functions: focus on adult humans. Exp Neurol.

[5] Rasmussen MK, Mestre H, Nedergaard M (2018). The glymphatic pathway in neurological disorders. Lancet Neurol.

[6] Brinker T, Stopa E, Morrison J, Klinge P (2014). A new look at cerebrospinal fluid circulation. Fluids Barriers CNS.

[7] Matsumae M, Kuroda K, Yatsushiro S, Hirayama A, Hayashi N, Takizawa K, Atsumi H, Sorimachi T (2019). Changing the currently held concept of cerebrospinal fluid dynamics based on shared findings of cerebrospinal fluid motion in the cranial cavity using various types of magnetic resonance imaging techniques. Neurol Med Chir (Tokyo).

[8] Klarica M, Radoš M, Orešković D (2019). The movement of cerebrospinal fluid and its relationship with substances behavior in cerebrospinal and interstitial fluid. Neuroscience.

[9] Yamada S, Tsuchiya K, Bradley WG, Law M, Winkler ML, Borzage MT, Miyazaki M, Kelly EJ, McComb JG (2015). Current and emerging MR imaging techniques for the diagnosis and management of CSF flow disorders: a review of phase-contrast and time-spatial labeling inversion pulse. AJNR Am J Neuroradiol.

[10] Cushing HW, Milford H (1925). The third circulation and its channels. Studies in intracranial physiology & surgery: the third circulation, the hypophysics, the gliomas.

[11] Oreskovic D, Rados M, Klarica M (2016). Cerebrospinal fluid secretion by the choroid plexus?. Physiol Rev.

[12] Eide PK, Valnes LM, Pripp AH, Mardal KA, Ringstad G (2020). Delayed clearance of cerebrospinal fluid tracer from choroid plexus in idiopathic normal pressure hydrocephalus. J Cereb Blood Flow Metab.

[13] Eide PK, Pripp AH, Ringstad G (2020). Magnetic resonance imaging biomarkers of cerebrospinal fluid tracer dynamics in idiopathic normal pressure hydrocephalus. Brain Commun.

[14] Ringstad G, Vatnehol SAS, Eide PK (2017). Glymphatic MRI in idiopathic normal pressure hydrocephalus. Brain.

[15] Ringstad G, Valnes LM, Dale AM, Pripp AH, Vatnehol SS, Emblem KE, Mardal KA, Eide PK (2018). Brain-wide glymphatic enhancement and clearance in humans assessed with MRI. JCI Insight.

[16] Brix MK, Westman E, Simmons A, Ringstad GA, Eide PK, Wagner-Larsen K, Page CM, Vitelli V, Beyer MK (2017). The Evans’ Index revisited: new cut-off levels for use in radiological assessment of ventricular enlargement in the elderly. Eur J Radiol.

[17] Virhammar J, Laurell K, Cesarini KG, Larsson EM (2014). The callosal angle measured on MRI as a predictor of outcome in idiopathic normal-pressure hydrocephalus. J Neurosurg.

[18] Hashimoto M, Ishikawa M, Mori E, Kuwana N (2010). Diagnosis of idiopathic normal pressure hydrocephalus is supported by MRI-based scheme: a prospective cohort study. Cerebrospinal Fluid Res.

[19] Fischl B (2012). FreeSurfer. Neuroimage.

[20] Lindstrom EK, Ringstad G, Mardal KA, Eide PK (2018). Cerebrospinal fluid volumetric net flow rate and direction in idiopathic normal pressure hydrocephalus. NeuroImage Clin.

[21] Ringstad G, Lindstrom EK, Vatnehol SAS, Mardal KA, Emblem KE, Eide PK (2017). Non-invasive assessment of pulsatile intracranial pressure with phase-contrast magnetic resonance imaging. PLoS ONE.

[22] Baledent O, Gondry-Jouet C, Meyer ME, De Marco G, Le Gars D, Henry-Feugeas MC, Idy-Peretti I (2004). Relationship between cerebrospinal fluid and blood dynamics in healthy volunteers and patients with communicating hydrocephalus. Invest Radiol.

[23] Wagshul ME, Chen JJ, Egnor MR, McCormack EJ, Roche PE (2006). Amplitude and phase of cerebrospinal fluid pulsations: experimental studies and review of the literature. J Neurosurg.

[24] Eide PK, Sorteberg W (2010). Diagnostic intracranial pressure monitoring and surgical management in idiopathic normal pressure hydrocephalus: a 6-year review of 214 patients. Neurosurgery.

[25] Eide PK, Sorteberg W (2016). Outcome of surgery for idiopathic normal pressure hydrocephalus: role of preoperative static and pulsatile intracranial pressure. World Neurosurg.

[26] Farb RI, Nicholson PJ, Peng PW, Massicotte EM, Lay C, Krings T, terBrugge KG (2019). Spontaneous intracranial hypotension: a systematic imaging approach for CSF leak localization and management based on MRI and digital subtraction myelography. AJNR Am J Neuroradiol.

[27] Horie T, Kajihara N, Matsumae M, Obara M, Hayashi N, Hirayama A, Takizawa K, Takahara T, Yatsushiro S, Kuroda K (2017). Magnetic resonance imaging technique for visualization of irregular cerebrospinal fluid motion in the ventricular system and subarachnoid space. World Neurosurg.

[28] Mollan SP, Davies B, Silver NC, Shaw S, Mallucci CL, Wakerley BR, Krishnan A, Chavda SV, Ramalingam S, Edwards J (2018). Idiopathic intracranial hypertension: consensus guidelines on management. J Neurol Neurosurg Psychiatry.

[29] Ringstad G, Eide PK (2020). Cerebrospinal fluid tracer efflux to parasagittal dura in humans. Nat Commun.

[30] Eide PK (2016). The correlation between pulsatile intracranial pressure and indices of intracranial pressure–volume reserve capacity: results from ventricular infusion testing. J Neurosurg.

[31] Lindstrom EK, Ringstad G, Sorteberg A, Sorteberg W, Mardal KA, Eide PK (2019). Magnitude and direction of aqueductal cerebrospinal fluid flow: large variations in patients with intracranial aneurysms with or without a previous subarachnoid hemorrhage. Acta Neurochir (Wien).

[32] Gatehouse PD, Rolf MP, Graves MJ, Hofman MB, Totman J, Werner B, Quest RA, Liu Y, von Spiczak J, Dieringer M (2010). Flow measurement by cardiovascular magnetic resonance: a multi-centre multi-vendor study of background phase offset errors that can compromise the accuracy of derived regurgitant or shunt flow measurements. J Cardiovasc Magn Reson.

[33] Penn RD, Basati S, Sweetman B, Guo X, Linninger A (2011). Ventricle wall movements and cerebrospinal fluid flow in hydrocephalus. J Neurosurg.

[34] Ringstad G, Emblem KE, Eide PK (2016). Phase-contrast magnetic resonance imaging reveals net retrograde aqueductal flow in idiopathic normal pressure hydrocephalus. J Neurosurg.

[35] Bateman GA, Brown KM (2012). The measurement of CSF flow through the aqueduct in normal and hydrocephalic children: from where does it come, to where does it go?. Childs Nerv Syst.

[36] Kim DS, Choi JU, Huh R, Yun PH, Kim DI (1999). Quantitative assessment of cerebrospinal fluid hydrodynamics using a phase-contrast cine MR image in hydrocephalus. Childs Nerv Syst.

[37] Yin LK, Zheng JJ, Zhao L, Hao XZ, Zhang XX, Tian JQ, Zheng K, Yang YM (2017). Reversed aqueductal cerebrospinal fluid net flow in idiopathic normal pressure hydrocephalus. Acta Neurol Scand.

[38] Cogswell PM, Graff-Radford J, Wurtz LI, Graff-Radford NR, Johnson DR, Hunt CH, Gunter JL, Cutsforth-Gregory JK, Jones DT, Eleder BD, Huston III J, Jack CR (2020). CSF dynamics disorders: association of brain MRI and nuclear medicine cisternogram findings. NeuroImage Clin.

[39] Graff-Radford J, Gunter JL, Jones DT, Przybelski SA, Schwarz CG, Huston J, Lowe V, Elder BD, Machulda MM, Gunter NB (2019). Cerebrospinal fluid dynamics disorders: relationship to Alzheimer biomarkers and cognition. Neurology.

[40] Haga PT, Pizzichelli G, Mortensen M, Kuchta M, Pahlavian SH, Sinibaldi E, Martin BA, Mardal KA (2017). A numerical investigation of intrathecal isobaric drug dispersion within the cervical subarachnoid space. PLoS ONE.

[41] Trillo-Contreras JL, Toledo-Aral JJ, Echevarría M, Villadiego J (2019). AQP1 and AQP4 contribution to cerebrospinal fluid homeostasis. Cells.

[42] Lam MA, Hemley SJ, Najafi E, Vella NGF, Bilston LE, Stoodley MA (2017). The ultrastructure of spinal cord perivascular spaces: implications for the circulation of cerebrospinal fluid. Sci Rep.

[43] Bedussi B, van der Wel NN, de Vos J, van Veen H, Siebes M, VanBavel E, Bakker EN (2017). Paravascular channels, cisterns, and the subarachnoid space in the rat brain: a single compartment with preferential pathways. J Cereb Blood Flow Metab.

[44] Sonnenberg H, Solomon S, Frazier DT (1967). Sodium and chloride movement into the central canal of cat spinal cord. Proc Soc Exp Biol Med.

[45] Kajimoto Y, Ohta T, Miyake H, Matsukawa M, Ogawa D, Nagao K, Kuroiwa T (2000). Posture-related changes in the pressure environment of the ventriculoperitoneal shunt system. J Neurosurg.

[46] Spijkerman JM, Geurts LJ, Siero JCW, Hendrikse J, Luijten PR, Zwanenburg JJM (2018). Phase contrast MRI measurements of net cerebrospinal fluid flow through the cerebral aqueduct are confounded by respiration. J Magn Reson Imaging.

[47] Vinje V, Ringstad G, Lindstrom EK, Valnes LM, Rognes ME, Eide PK, Mardal KA (2019). Respiratory influence on cerebrospinal fluid flow—a computational study based on long-term intracranial pressure measurements. Sci Rep.

[48] Dreha-Kulaczewski S, Joseph AA, Merboldt KD, Ludwig HC, Gartner J, Frahm J (2017). Identification of the upward movement of human CSF in vivo and its relation to the brain venous system. J Neurosci.

[49] Aktas G, Kollmeier JM, Joseph AA, Merboldt KD, Ludwig HC, Gärtner J, Frahm J, Dreha-Kulaczewski S (2019). Spinal CSF flow in response to forced thoracic and abdominal respiration. Fluids Barriers CNS.

[50] Nilsson C, Stahlberg F, Thomsen C, Henriksen O, Herning M, Owman C (1992). Circadian variation in human cerebrospinal fluid production measured by magnetic resonance imaging. Am J Physiol.

[51] Sartoretti T, Wyss M, Sartoretti E, Reischauer C, Hainc N, Graf N, Binkert C, Najafi A, Sartoretti-Schefer S (2019). Sex and age dependencies of aqueductal cerebrospinal fluid dynamics parameters in healthy subjects. Front Aging Neurosci.

[52] Bering EA (1952). Water exchange of central nervous system and cerebrospinal fluid. J Neurosurg.

[53] Edeklev CS, Halvorsen M, Lovland G, Vatnehol SAS, Gjertsen O, Nedregaard B, Sletteberg R, Ringstad G, Eide PK (2019). Intrathecal use of gadobutrol for glymphatic MR imaging: prospective safety study of 100 patients. AJNR Am J Neuroradiol.

[54] Halvorsen M, Edeklev CS, Fraser-Green J, Lovland G, Vatnehol SAS, Gjertsen O, Nedregaard B, Sletteberg R, Ringstad G, Eide PK (2021). Off-label intrathecal use of gadobutrol: safety study and comparison of administration protocols. Neuroradiology.

[55] Patel M, Atyani A, Salameh JP, McInnes M, Chakraborty S (2020). Safety of intrathecal administration of gadolinium-based contrast agents: a systematic review and meta-analysis. Radiology.

[56] Nitz WR, Bradley WG, Watanabe AS, Lee RR, Burgoyne B, O'Sullivan RM, Herbst MD (1992). Flow dynamics of cerebrospinal fluid: assessment with phase-contrast velocity MR imaging performed with retrospective cardiac gating. Radiology.

