# Supplementary material for: Direction and magnitude of cerebrospinal fluid flow vary substantially across central nervous system diseases
Source: Fluids Barriers CNS. 2021 Apr 1;18:16. doi: 10.1186/s12987-021-00251-6 (PMC8017867; doi:10.1186/s12987-021-00251-6)
Supplement: Supplementary file 1 — Additional file 1: Figure S1: Examples of phase-contrast MRI of the cerebral aqueduct. For different patients, the regions of interest within the CSF of the cerebral aqueduct is shown in red and the reference regions of interest in blue (a, c, e, g). The CSF flow velocity is shown for the different pixels with mean flow velocity of all pixels indicated by a dark line, including noise level of pixels in reference tissue (colored lines) with mean noise level indicated by dark stippled line (b, d, f, h). The velocities from each pixel were assessed using MATLAB, and expressed as centimeters per second. Positive values refer to cranial CSF flow direction and negative values caudal CSF flow direction. Figure S2: Examples of phase-contrast MRI of the cranio-cervical junction. For different patients, the regions of interest within the CSF of the cranio-cervical junction is shown in red and the reference regions of interest in blue (a, c). The CSF flow velocity is shown for the different pixels with mean flow velocity of all pixels indicated by a dark line, including noise level of pixels in reference tissue (colored lines) with mean noise level indicated by dark stippled line (b, d). The velocities from each pixel were assessed using MATLAB, and expressed as centimeters per second. Positive values refer to cranial CSF flow direction and negative values caudal CSF flow direction. Figure S3: Association between the mean ICP wave amplitude (MWA) and enrichment of tracer within third and lateral ventricles. There was a significant positive correlation between the mean ICP wave amplitude (MWA) measured over-night and tracer enrichment within 3rd ventricle after 6 hours (a; n=47) and 24 hours (b; n=50), and between MWA and tracer enrichment within lateral ventricles after 6 hours (c; n=47) and 24 hours (d; n=50). Each plot shows the fit line with Pearson correlation coefficients and significance levels. Figure S4: Association between the callosal angle and enrichment of tracer w [file 12987_2021_251_MOESM1_ESM.pdf]

Additional file 1

Direction and magnitude of cerebrospinal fluid flow vary substantially across central nervous system diseases

Per Kristian Eide, MD, PhD[1,2], Lars Magnus Valnes, PhD[1], Erika Kristina Lindstrøm, PhD[3,4], Kent-Andre Mardal, PhD[3,5], Geir Ringstad, MD, PhD[6]

[1]*Deptartment of Neurosurgery, Oslo University Hospital-Rikshospitalet, Oslo, Norway*

[2]*Institute of Clinical Medicine, Faculty of Medicine, University of Oslo, Oslo, Norway,*

[3]*Department of Mathematics, Faculty of Mathematics and Natural Sciences, University of Oslo, Norway.*

[4]*Institute for cancer genetics and informatics, Oslo University hospital*

[5]*Department of Numerical Analysis and Scientific Computing, Simula Research Laboratory, Oslo, Norway;*

[6]*Department. of Radiology, Oslo University Hospital- Rikshospitalet, Oslo, Norway.*

Corresponding author:

Professor Per Kristian Eide, MD PhD

Dept. of Neurosurgery,

Oslo University Hospital – Rikshospitalet,

PB 4950 Nydalen, 0424 OSLO, Norway

Phone: +47 91649419; Fax: +47-23074310

E-mail: p.k.eide@medisin.uio.no

Supplementary Figure 1

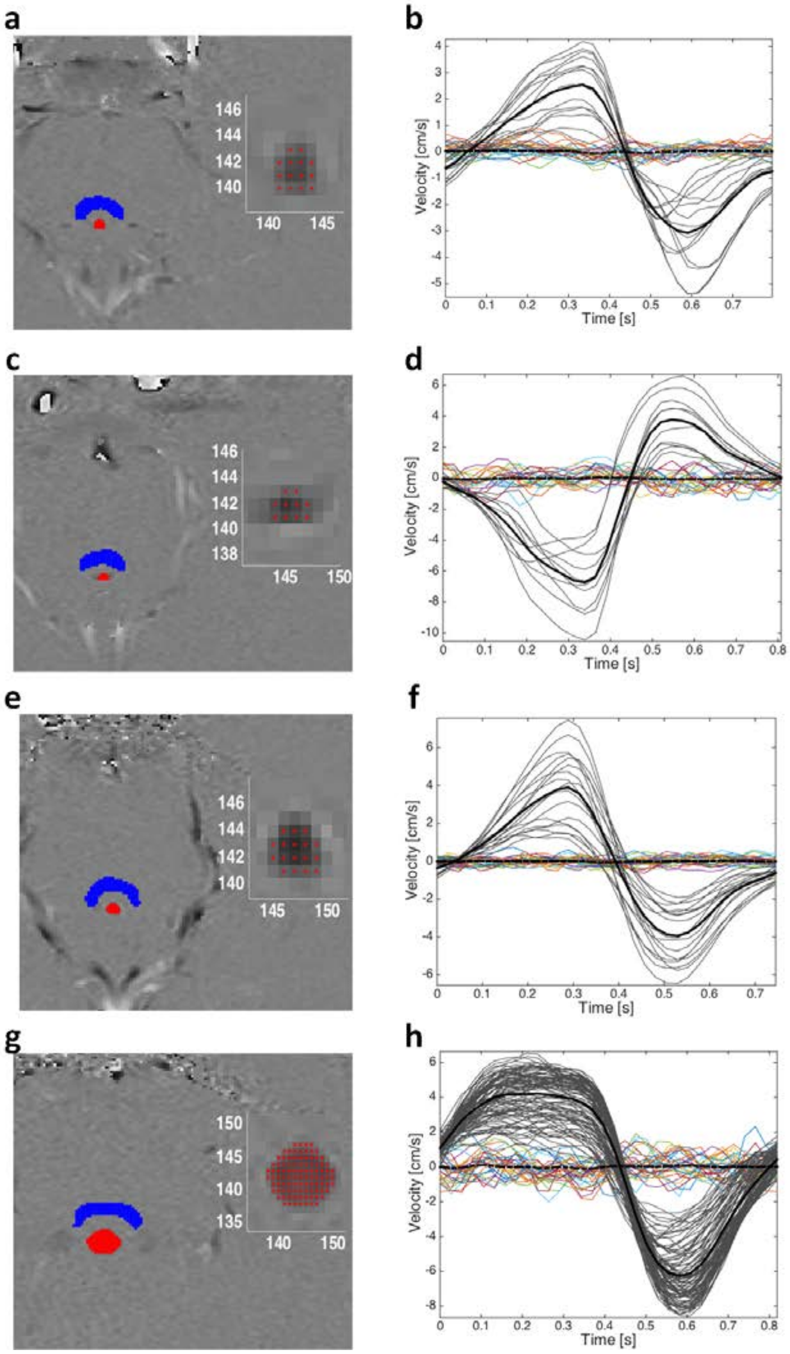

Examples of phase-contrast MRI of the cerebral aqueduct. For different patients, the regions of interest within the CSF of the cerebral aqueduct is shown in red and the reference regions of interest in blue (a, c, e, g). The CSF flow velocity is shown for the different pixels with mean flow velocity of all pixels indicated by a dark line, including noise level of pixels in reference tissue (colored lines) with mean noise level indicated by dark stippled line (b, d, f, h). The velocities from each pixel were assessed using MATLAB, and expressed as centimeters per second. Positive values refer to cranial CSF flow direction and negative values caudal CSF flow direction.

Supplementary Figure 2

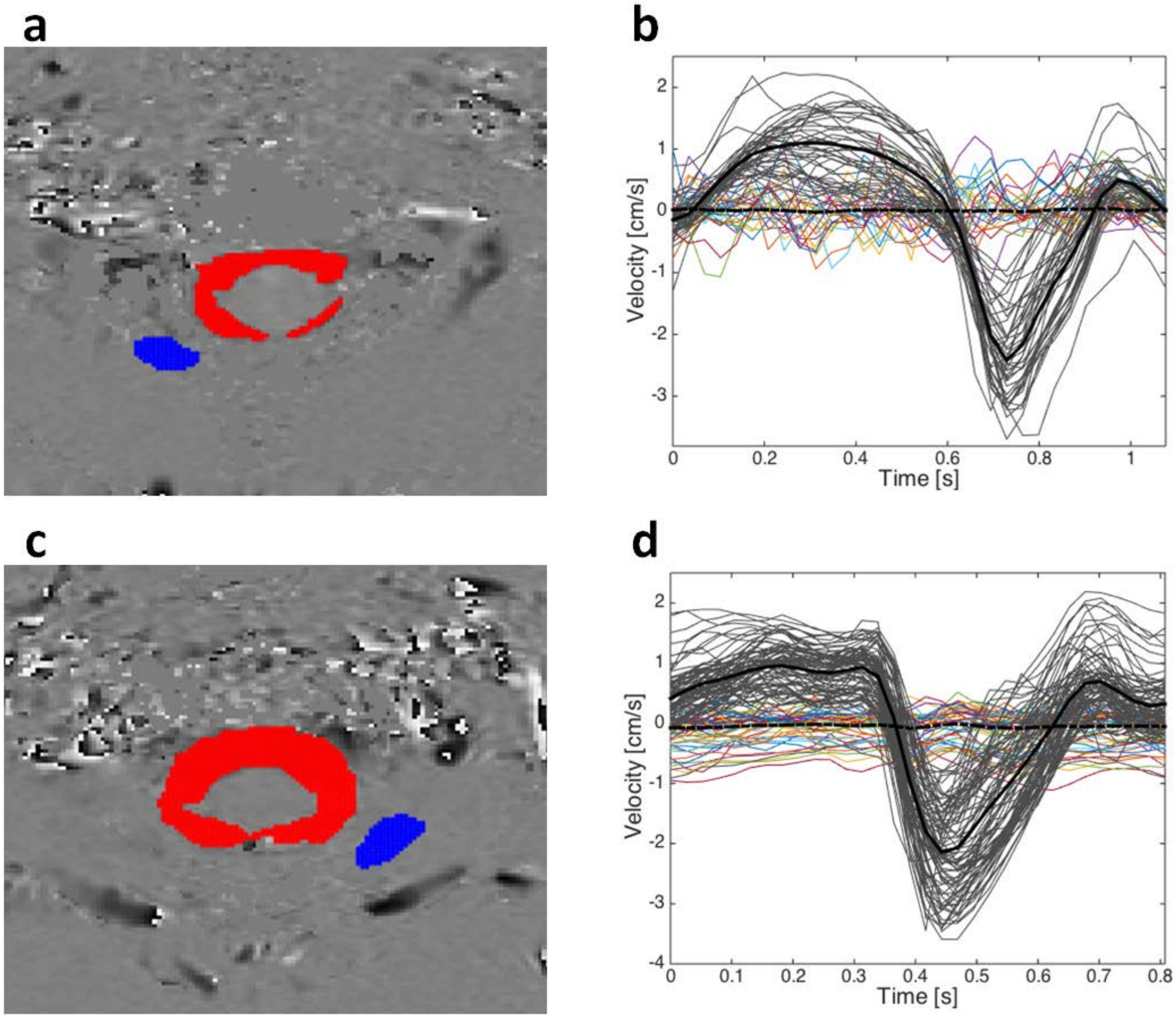

Examples of phase-contrast MRI of the cranio-cervical junction. For different patients, the regions of interest within the CSF of the cranio-cervical junction is shown in red and the reference regions of interest in blue (a, c). The CSF flow velocity is shown for the different pixels with mean flow velocity of all pixels indicated by a dark line, including noise level of pixels in reference tissue (colored lines) with mean noise level indicated by dark stippled line (b, d). The velocities from each pixel were assessed using MATLAB, and expressed as centimeters per second. Positive values refer to cranial CSF flow direction and negative values caudal CSF flow direction.

Supplementary Figure 3

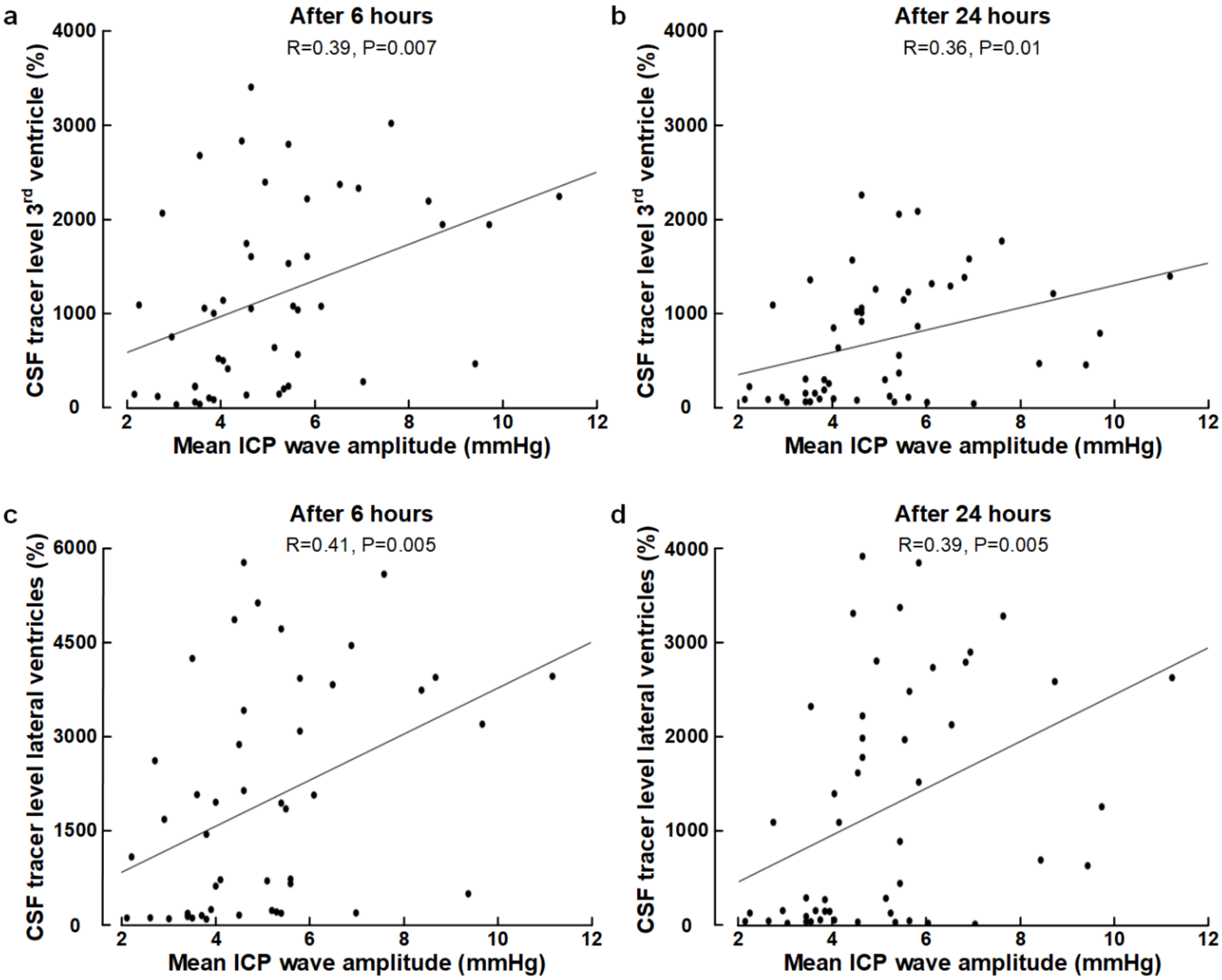

Association between the mean ICP wave amplitude (MWA) and enrichment of tracer within third and lateral ventricles. There was a significant positive correlation between the mean ICP wave amplitude (MWA) measured over-night and tracer enrichment within 3rd ventricle after 6 hours (a; n=47) and 24 hours (b; n=50), and between MWA and tracer enrichment within lateral ventricles after 6 hours (c; n=47) and 24 hours (d; n=50). Each plot shows the fit line with Pearson correlation coefficients and significance levels.

Supplementary Figure 4

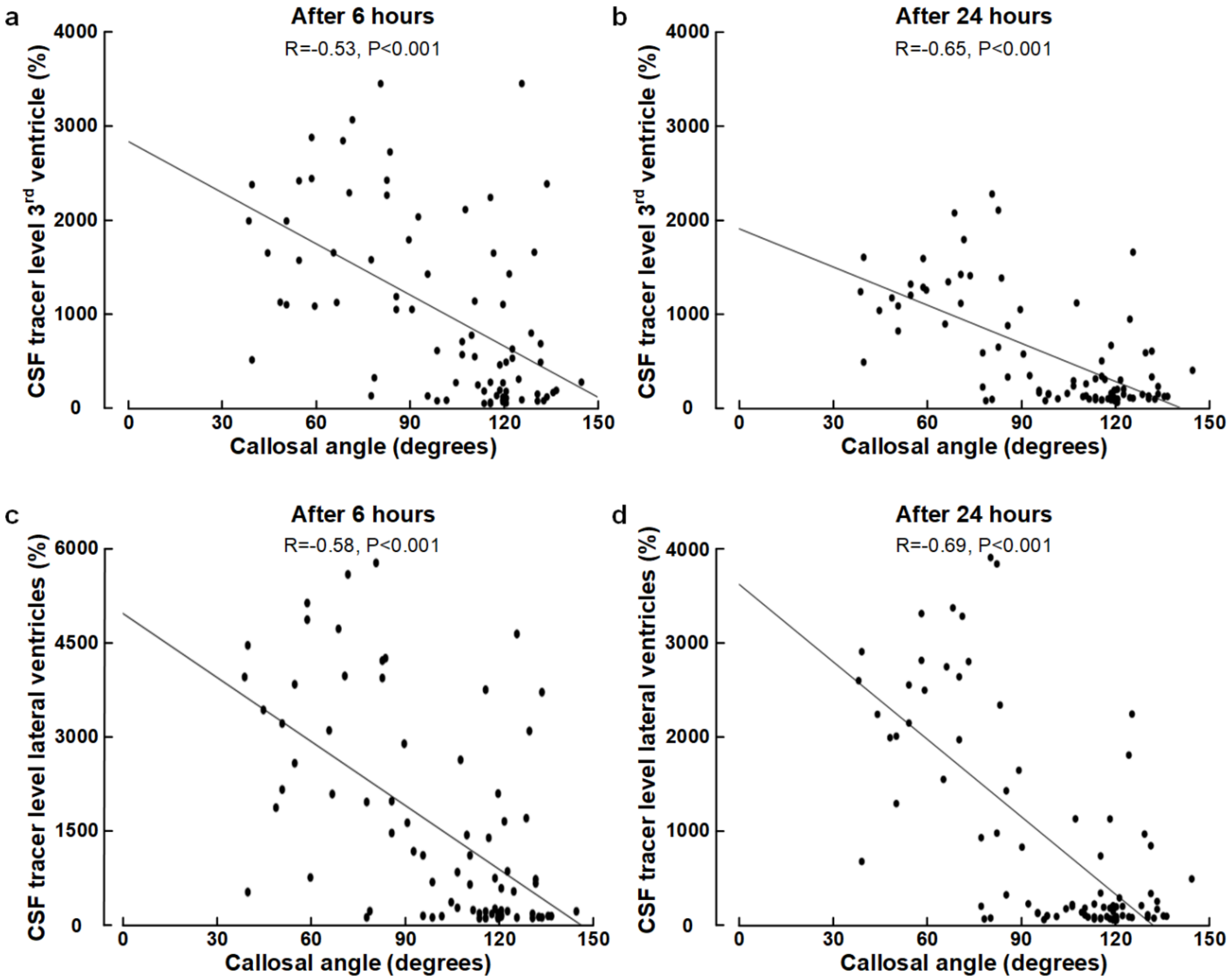

Association between the callosal angle and enrichment of tracer within third and lateral ventricles. There was a significant negative correlation between the callosal angle and tracer enrichment within 3rd ventricle after 6 hours (a; n=81) and 24 hours (b; n=88), and between callosal angle and tracer enrichment within lateral ventricles after 6 hours (c; n=81) and 24 hours (d; n=88). Each plot shows the fit line with Pearson correlation coefficients and significance levels.

Supplementary Figure 5

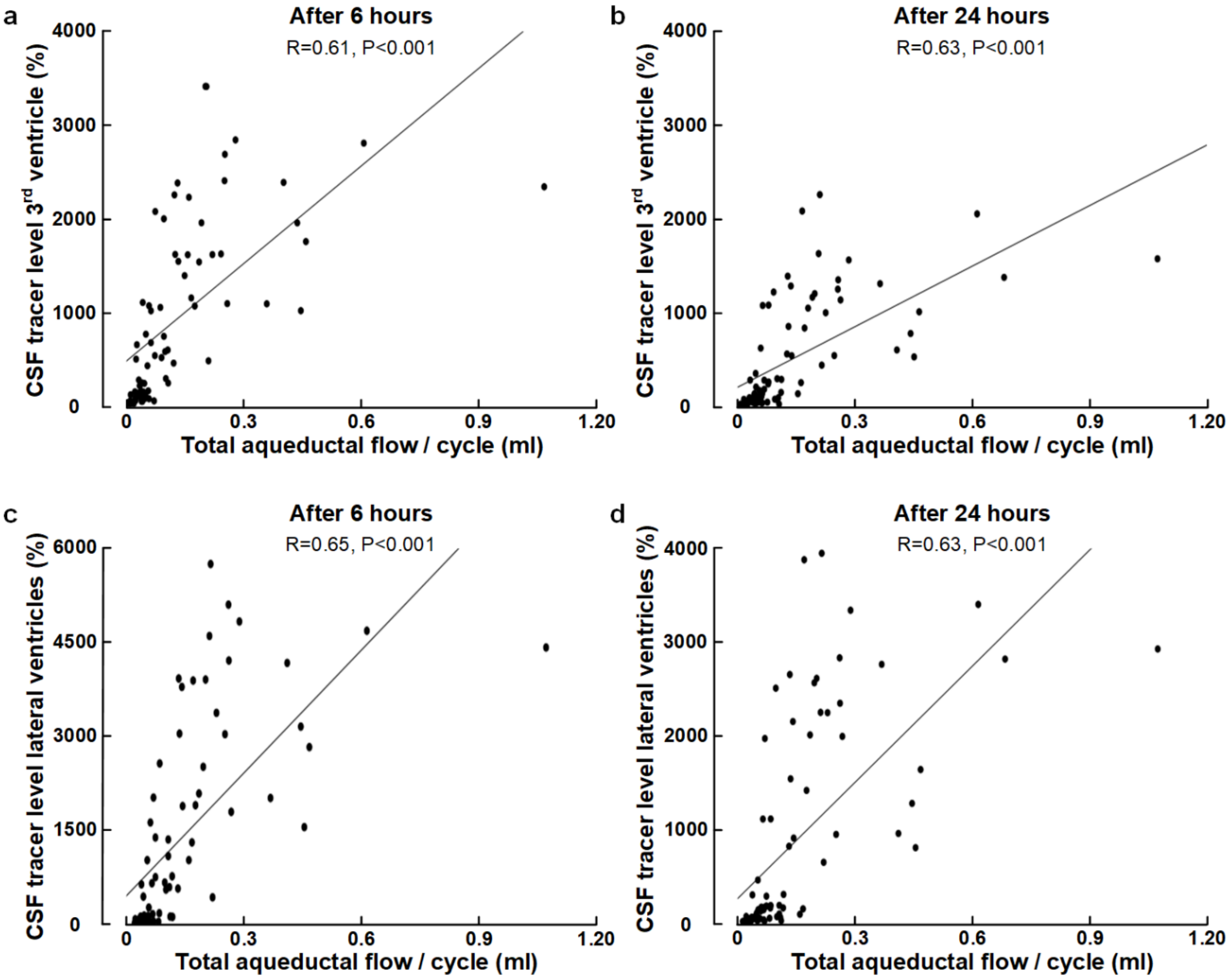

Association between the total aqueductal flow per cycle and enrichment of tracer within third and lateral ventricles. There was a significant positive correlation between the total aqueductal flow per cycle and tracer enrichment within 3rd ventricle after 6 hours (a; n=74) and 24 hours (b; n=80), and between total aqueductal flow per cycle and tracer enrichment within lateral ventricles after 6 hours (c; n=74) and 24 hours (d; n=80). Each plot shows the fit line with Pearson correlation coefficients and significance levels.

Supplementary Figure 6

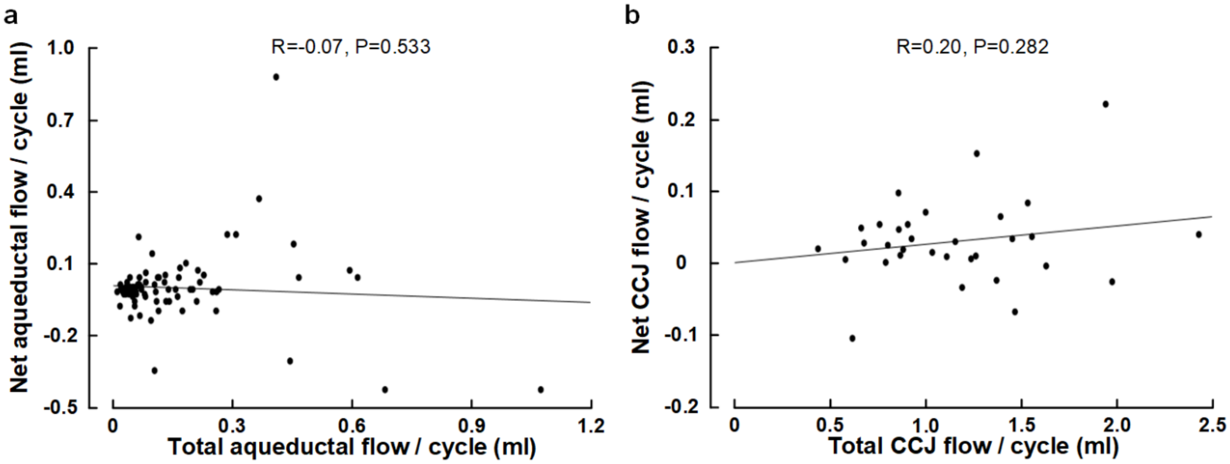

Association between the total flow and net flow per cycle in cerebral aqueduct and craniocervical junction. There was no correlation between the total flow and net flow per cycle in (a) the cerebral aqueduct (n=85) and (b) the cranio-cervical junction (n=32). Each plot shows the fit line with Pearson correlation coefficients and significance levels.

Supplementary Figure 7

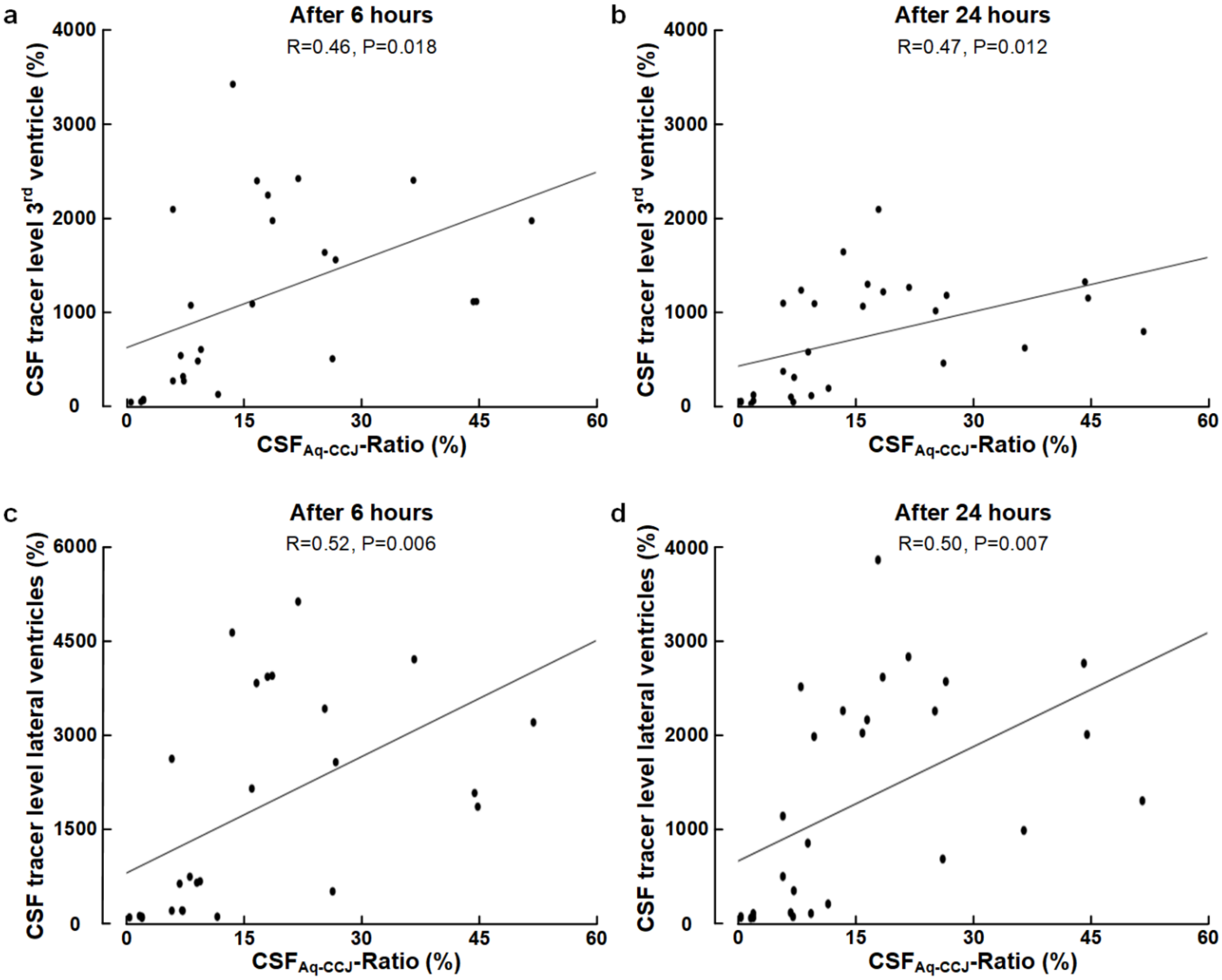

Association between the $CSF_{Aq\text{-}CCJ}$-Ratio and enrichment of tracer within third and lateral ventricles. There was a significant positive correlation between the $CSF_{Aq\text{-}CCJ}$-Ratio and tracer enrichment within 3rd ventricle after 6 hours (a; n=26) and 24 hours (b; n=28), and between the $CSF_{Aq\text{-}CCJ}$-Ratio and tracer enrichment within lateral ventricles after 6 hours (c; n=26) and 24 hours (d; n=28). Each plot shows the fit line with Pearson correlation coefficients and significance levels.

Supplementary Figure 8

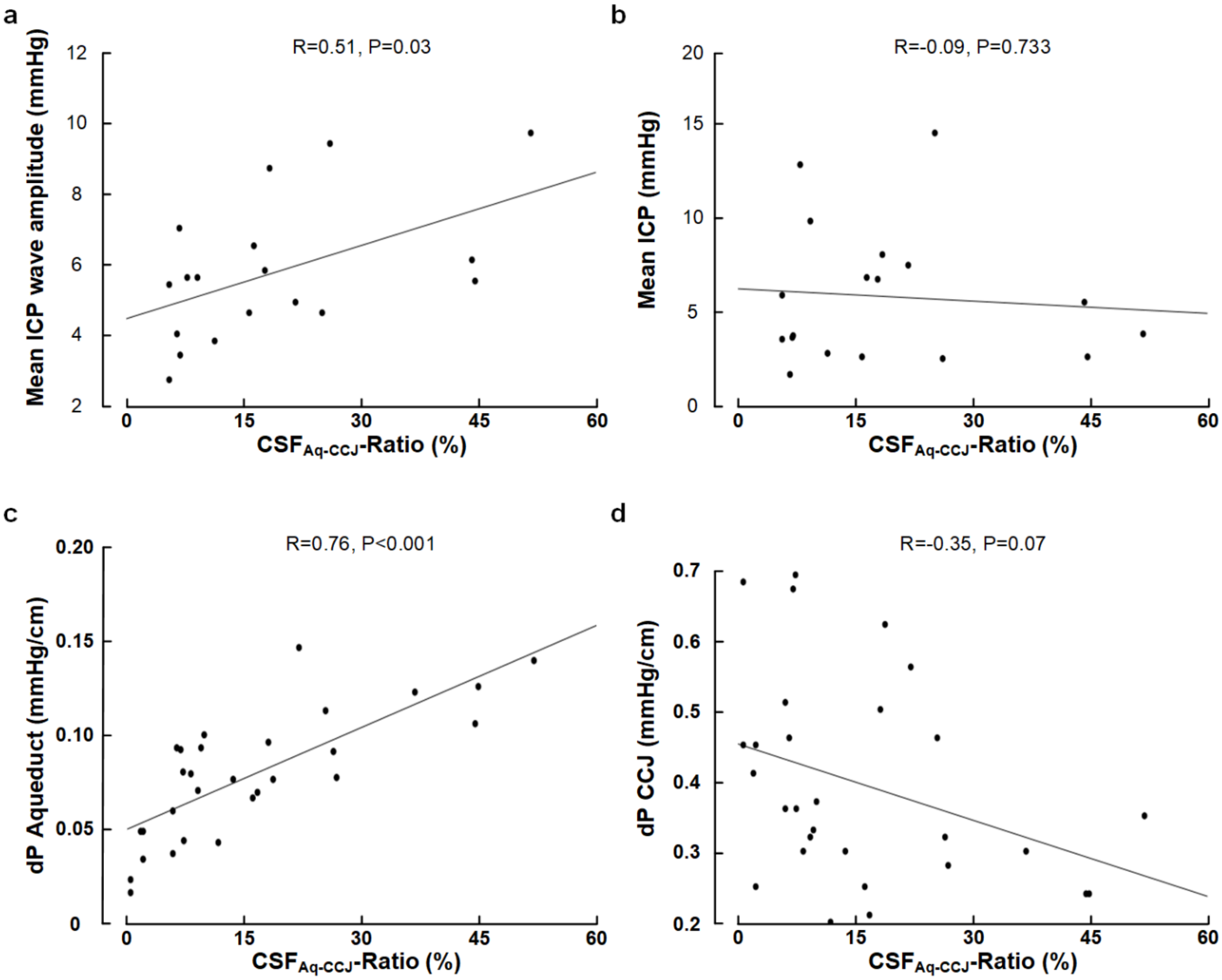

Association between the CSF_{Aq-CCJ}-Ratio and the mean ICP wave amplitude (MWA and mean ICP, and between the CSF_{Aq-CCJ}-Ratio and the pressure gradient in the cerebral aqueduct and cranio-cervical junction. There was a significant positive correlation between the CSF_{Aq-CCJ}-Ratio and the mean ICP wave amplitude (a; n=18) but no correlation with the mean ICP (b; n=18). The CSF_{Aq-CCJ}-Ratio was positively correlated with the pressure gradient (dP) at the cerebral aqueduct (c; n=29), but not with the pressure gradient (dP) in the craniocervical junction (d; n=29). Each plot shows the fit line with Pearson correlation coefficients and significance levels.

Supplementary Figure 9

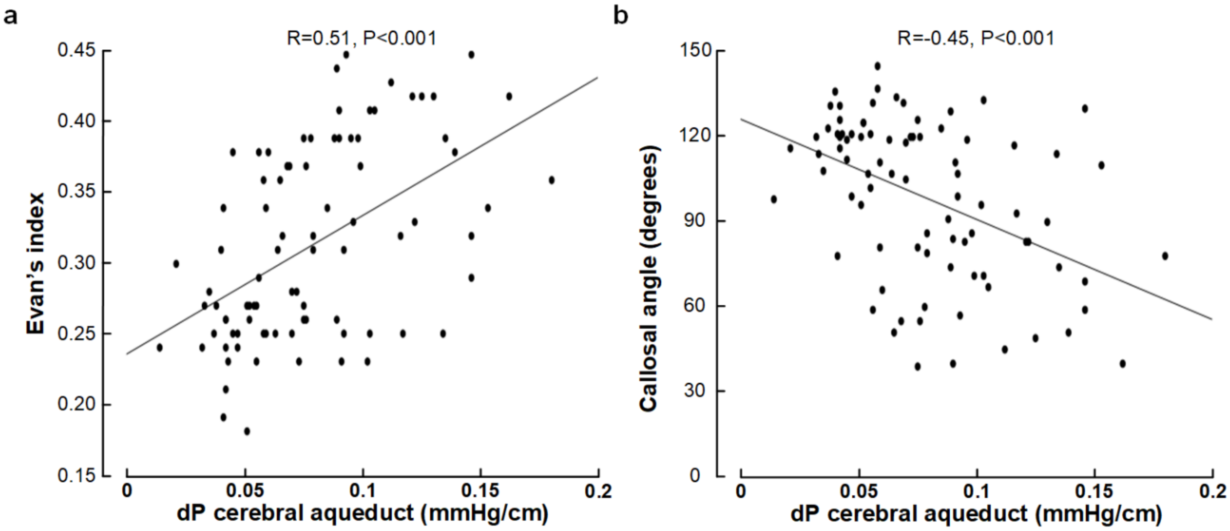

Association between the pressure gradient in the cerebral aqueduct and the biomarkers of CSF space Evan's index and callosal angle. There was a significant positive correlation between the pressure gradient (dP) at the cerebral aqueduct and the Evan's index (a; n=85), and a significant negative correlation between the pressure gradient (dP) at the cerebral aqueduct and the callosal angle (b; n=85). Each plot shows the fit line with Pearson correlation coefficients and significance levels.
